# Chemical Characterization of Red Wine Polymers and Their Interaction Affinity with Odorants

**DOI:** 10.3390/foods13040526

**Published:** 2024-02-08

**Authors:** Anna Maria Gabler, Annalena Ludwig, Florian Biener, Magdalena Waldner, Corinna Dawid, Oliver Frank

**Affiliations:** 1 Chair of Food Chemistry and Molecular Sensory Science, TUM School of Life Sciences, Technical University of Munich, Lise-Meitner-Str. 34, D-85354 Freising, Germany; annamaria.gabler@tum.de (A.M.G.); annalenaludwig@gmx.de (A.L.);; 2 Professorship for Functional Phytometabolomics, TUM School of Life Sciences, Technical University of Munich, Lise-Meitner-Str. 34, D-85354 Freising, Germany

**Keywords:** red wine, aroma binding, NMR spectroscopy, qHNMR, red wine polymers, high molecular weight, chemical degradation, sensory analysis

## Abstract

In order to characterize red wine polymers with regard to their binding properties to aroma compounds (odorants), a qualitative and quantitative analysis of chemical degradation products after different chemical treatments (thiolytic, acidic, and alkaline depolymerization) of high -molecular-weight (HMW) fractions of red wine was performed. Using ^1^H NMR, LC-ToF-MS, LC-MS/MS, and HPIC revealed key structural features such as carbohydrates, organic acids, phenolic compounds, anthocyanins, anthocyanidins, amino acids, and flavan-3-ols responsible for odorant-polymer interactions. Further, NMR-based interaction studies of the selected aroma compounds 3-methylbutanol, *cis*-whisky lactone, 3-methylbutanoic acid, and 3-isobutyl-2-methoxypyrazine with HMW polymers after chemical treatment demonstrated a reduced interaction affinity of the polymer compared to the native HMW fractions, and further, the importance of aromatic compounds such as flavan-3-ols for the formation of odorant polymer interactions. In addition, these observations could be verified by human sensory experiments. For the first time, the combination of a compositional analysis of red wine polymers and NMR-based interaction studies with chemically treated HMW fractions enabled the direct analysis of the correlation of the polymer’s structure and its interaction affinity with key odorants in red wine.

## 1. Introduction

The unique and desirable flavor of red wine attracts consumers every day all over the world. This wine flavor was analyzed in numerous studies [1,2,3,4,5,6] and suggested to be highly influenced by a nonvolatile matrix [3,5,7,8,9,10]. Besides single compounds such as sugars [11,12,13], anthocyanins [14], or polyphenols [15,16,17,18,19,20], polymers [21,22] were also shown to interact with sensory active molecules in red wine, which led to differences in their sensory profiles. By application of quantitative proton nuclear magnetic resonance spectroscopy (^1^H NMR), combined with human sensory studies, odorant and tastant polymer interactions were investigated in red wine for the first time, and, further, the gap in the flavor perception of aroma and taste reconstitution models and real red wine was bridged recently [21,22]. The NMR interaction studies of key aroma and taste active compounds and high-molecular-weight (HMW) fractions revealed different molecular interaction types in red wine depending on the structural properties of the odorants and tastants. The following interaction scenarios were observed for aroma and taste active compounds based on chemical shifts in the ^1^H NMR spectrum: (i) no shifts/interactions for amino acids, alcohols, carbohydrates, diketones, ellagitannins, esters, flavon-3-ol glycosides, furanones, and C_13_-norisoprenoids; (ii) shielded signals for lactones, aldehydes, phenolic acid ethyl esters, and flavan-3-ols; (iii) de-shielded signals for acids, alditols, and salts; and (iv) signal shifts to lower frequencies and signal changes typical for noncovalent π–π interactions for pyrazines and phenols [21,22].

Although the data on the location of interaction sites in the red wine polymer are rather fragmentary, single constituents of HMW polymers have been analyzed after different chemical degradation methods (alkaline and acidic hydrolysis and thiolytic depolymerization) using several analytical approaches, such as HPLC-UV/Vis or LC-MS/MS in the past. The studies revealed a procyanidin backbone of the red wine polymer containing flavan-3-ol derivatives, linked to organic acids, phenolic acids, carbohydrates, polyols, amino acids, and anthocyanins, respectively [23,24]. To better understand the formation of interactions of the red wine polymer, Koch et al., (2023) [25] developed a new approach using density functional theory (DFT) calculations, NMR experiments, and human sensory studies to evaluate potential interaction sites of aroma active compounds in the red wine polymer. Therefore, the interaction affinity of four odorants (3-methylbutanol, 3-methylbutanoic acid, *cis*-whisky lactone, and 3-isobutyl-2-methoxypyrazine), each representing one interaction scenario, and specific polymer segments ((+)-catechin, *p*-coumaric acid, aspartic acid, tartaric acid, and galactose), each representing one structural class of degradation products, were analyzed. The studies showed the highest interaction affinities and even recognizable sensory effects between 3-isobutyl-2-methoxypyrazine and (+)-catechin and *p*-coumaric acid, respectively, as well as between *cis*-whisky lactone and (+)-catechin. In detail, 3-isobutyl-2-methoxypyrazine showed π–π interactions with (+)-catechin and *p*-coumaric acid, whereas *cis*-whisky lactone revealed van der Waals interactions with the aromatic compounds. The analytes 3-methylbutanol and 3-methylbutanoic acid showed the lowest interaction affinity with the aromatic polymer segments and aspartic acid. Tartaric acid, representing the group of organic acids in the polymer, demonstrated a high interaction affinity to the pyrazine, followed by 3-methylbutanol, 3-methylbutanoic acid, and *cis*-whisky lactone. Galactose formed hydrogen bonds with 3-methylbutanol and 3-methylbutanoic acid and weak van der Waals interactions with the remaining odorants. Among the polymer segments, the aromatic substances (+)-catechin and *p*-coumaric acid showed the strongest binding energies, and further, the most stable conformations [25].

Although the literature data gave a first insight into odorant and tastant polymer interactions and their influence on the flavor of red wine, a direct correlation between the molecular interaction type and the constitution of the HMW polymer has not yet been investigated. Further, the knowledge of possible interaction sites in the polymer and the molecular mechanism leading to polymer interactions is fragmentary. For this reason, the objectives of the current study were as follows:

(1) To identify and quantify products released from the red wine HMW fractions after different chemical treatments using UHPLC-ToF-MS, ^1^H NMR spectroscopy, LC-MS/MS, and HPIC;
(2) To apply quantitative ^1^H NMR-based interaction studies to directly analyze the influence of chemical degradation products on the interaction activity of the polymer;
(3) To study the correlation between the chemical composition and the NMR interaction activity of red wine polymers.

## 2. Materials and Methods

### 2.1. Samples

The analyzed Primitivo red wine (14% vol ethanol, vintage 2019) was purchased from a local shop in Freising, Germany. Following a literature protocol [21], HMW red wine polymers were isolated using a Sartoflow Smart ultrafiltration system (Sartorius Stedim Biotech, Göttingen, Germany). Three different fractions (HMW > 50 kDa, 30–50 kDa, 5–30 kDa) were obtained and lyophilized for further experiments.

### 2.2. Chemicals

The compounds used for the present study are presented in the Supporting Information. Water for ultrafiltration, UHPLC-MS/MS and HPIC measurements was purified via a Milli-Q Reference A^+^ system (Millipore, Schwalbach, Germany). Solvents (acetonitrile, methanol) in LC-MS grade were purchased from Merck (Darmstadt, Germany). Stable isotope-labeled amino acids were obtained from Eurisotop (Saarbrücken, Germany).

### 2.3. Chemical Degradation Methods

#### 2.3.1. Acidic Hydrolysis with Sulfuric Acid

Using the Seaman approach with some modifications [23,26], an aliquot (0.45 mL) of sulfuric acid (72%, *w*/*w*) was added to aliquots (5 mg) of the HMW fractions and kept at room temperature for 2 h, respectively. Afterwards, the solution was diluted with water (5.8 mL) and heated for 2 h and 8 h at 100 °C, respectively. The reaction mixtures were cooled to room temperature and neutralized with aqueous sodium hydroxide solution (1 mol/L). For purification, the samples were washed with water (15 mL) using a water-conditioned C18 SPE-cartridge (1000 mg, Chromabond^®^ C_18_ ec, Machery-Nagel, Düren, Germany). The effluent was filled up with water (25 mL) and concentrated to 2.5 mL for quantitative measurements. The purification procedure was repeated for the native HMW fractions (5 mg in 2 mL water). The whole procedure was performed in triplicates for all HMW fractions.

#### 2.3.2. Acidic Hydrolysis with Hydrochloric Acid

Following a literature protocol [23], aliquots (5 mg) of HMW fractions were mixed with aqueous hydrochloric acid (6 mol/L; 3 mL) and heated for 24 h at 110 °C. The solution was then cooled in an ice-bath, neutralized with aqueous sodium hydroxide (12 mol/L), and made up with water (5 mL). The treatment with hydrochloric acid was conducted three times for all HMW fractions.

#### 2.3.3. Alkaline Hydrolysis

According to literature procedures [23,27], aliquots (10 mg) of lyophilized HMW fractions were dissolved in 1 mL of an alkaline solution (8 g sodium hydroxide, 1 g ascorbic acid, 1 mg ethylenediaminetetraacetic acid) and stirred for 2 h at 40 °C, 2 h at 60 °C, and 2 h at 80 °C, respectively. The solution was then cooled in an ice bath and neutralized with aqueous hydrochloric acid (1 mol/L). Afterwards, all samples were made with water (2 mL), and an aliquot was used for UHPLC-ToF-MS identification and LC-MS/MS quantitation of polyphenols and organic acids. All alkaline hydrolysis reactions were performed in triplicates for every fraction and reaction parameter.

#### 2.3.4. Thiolytic Degradation

Using literature protocols with some modifications [23,24,28,29,30], aliquots (5 mg) of HMW fractions were dissolved in methanol (0.5 mL) and mixed with an aliquot of methanolic hydrochloric acid (0.5 mL, 3.3% HCl in MeOH) and an aliquot of thiolytic reagent (1 mL, benzyl mercaptan 5% in MeOH). The mixture was heated at different reaction times and temperatures (2 min, 90 °C; 2 h 40 °C, 2 h 60 °C) while stirring. Further, each individual sample was put in an ice bath to stop the reaction, filled up to 2 mL with methanol/water (30/70, *v*/*v*), and used for the identification and quantitation of thiolytic depolymerization products by means of UHPLC-ToF-MS and LC-MS/MS. Triplicates of thiolytic degradations of all HMW fractions were performed.

### 2.4. Identification of Chemical Degradation Products of HMW Fractions by UPLC/Time-of-Flight Mass Spectrometry (LC-ToF-MS) Screenings

For the untargeted screening of the polymers for chemical degradation products, all approaches were mixed, resulting in one sample for each chemical treatment (alkaline hydrolysis, acidic hydrolysis with H_2_SO_4_, acidic hydrolysis with HCl, and thiolytic degradation). Aliquots (3 µL) of the solutions were injected into an Acquity UPLC core system (Waters; Manchester, UK) equipped with a binary solvent manager, a sample manager, and a column oven. Chromatographic separation was performed on a BEH C18 column (150 × 2 mm, 1.7 µm, 130 Å, Waters, Manchester, UK) at a flow rate of 0.4 mL/min and a temperature of 40 °C with aqueous formic acid as solvent A (0.1%, *v*/*v*) and formic acid in acetonitrile (0.1%, *v*/*v*) as solvent B using the following gradient: starting at 5% B, increasing the content of B to 100% within 4 min, isocratic elution at 100% B for 0.5 min, followed by decreasing the content of B to 5% within 0.2 min, and finally, holding at 5% B for another 0.3 min.

High-resolution mass spectra were acquired on a Synapt G2-S HDMS (Waters; Manchester, UK) in positive and negative ESI resolution mode. The capillary voltage was set at 2.5 kV and −2.0 kV, sampling cone at 19 V and 50 V, extraction cone at 4.0 kV, source temperature at 150 °C, desolvation temperature at 450 °C, cone gas at 30 L/h, and desolvation gas at 850 L/h. A solution of sodium formate (0.5 mmol/L) in isopropyl alcohol/H_2_O (9 + 1, *v*/*v*) was used for the calibration of the mass spectrometer. For auto-correction of the mass data, a solution of leucine enkephalin (1 ng/µL, *m*/*z* 556.2771 [M + H]^+^ and *m*/*z* 554.2615 [M − H]^−^) was used.

All data were processed with Progenisis QI 2.4 (Waters, Milford, MA, USA) and MassLynx 4.1 (Waters, Manchester, UK). In Progenisis QI, retention times were aligned using a randomly chosen QC injection, and samples were normalized to their total ion intensity. For peak picking an automatic peak picking limit, there was no retention time limit, and the following adducts were selected: [M + H]^+^ and [M]^+^ in the positive mode; [M − H]^−^, [2M − H]^−^, and [M − H_2_O − H]^−^ in the negative mode. A database containing structural MOL file information for 212 literature-known compounds in red wine [1,2,3,23,24,31,32,33,34,35,36,37] was created with Progenesis SDF Studio 1.0 (Waters, Milford, MA, USA) and applied to the HMW samples. The reference substances of the investigated compounds were analyzed in the same manner, and data were processed in MassLynx 4.1. Therefore, the *m/z* fragmentation and retention times of the HMW samples after chemical degradation were compared to the ones of the reference substances.

### 2.5. Ultra-High-Performance Liquid Chromatography–Triple Quadrupole Mass Spectrometry (UHPLC-MS/MS)

Mass spectrometric analysis was performed using four different LC-MS/MS systems that are presented in the Supporting Information.

All MS/MS systems were operated in the multiple reaction monitoring (MRM) mode. System 1 was used for the quantitation of amino acids, System 2 for the quantitation of polyphenols, System 3 for the quantitation of organic acids, and System 4 for the quantitation of anthocyanins and galacturonic acid, (*E*)-, and (*Z*)-aconitic acid. Data analysis was performed with the MultiQuant software (version 3.0.3; Sciex, Darmstadt, Germany).

The validation experiments for LC-MS/MS experiments regarding linearity, recovery rates, intraday as well as interday precision, limit of detection (LoD), and limit of quantitation (LoQ) are shown in Tables S1–S4. Therefore, the exact concentration of each standard compound stock solution was determined first by qHNMR. Ten calibration points were prepared by gradually diluting the stock solution one-to-two. Afterward, a linear regression of the MultiQuant software was applied. For recovery experiments, the standard compounds were spiked into HMW samples using three different concentration levels of the calibration curve as triplicates. Five spiked HMW samples were analyzed on the same day for intraday precision and on consecutive days for the interday precision. In both cases, the relative standard deviation was calculated. The calibration curve was further diluted and the signal-to-noise ratio was determined using the MultiQuant software for the determination of LOD (signal-to-noise ratio of 3) and LOQ values (signal-to-noise ratio of 10).

#### 2.5.1. Quantitative Analysis of Amino Acids

Quantitative analysis of the amino acids in the HMW fractions treated with hydrochloric acid was performed by a previously published LC-MS/MS method (system 1) using stable isotope dilution analysis (SIDA). For sample preparation, aliquots (990 μL) of hydrolyzed and non-treated HMW fractions were mixed with an aliquot (10 μL) of internal standard mix [3,23,38].

#### 2.5.2. Quantitative Analysis of Anthocyanins

For the quantitation of anthocyanins, aliquots (1 µL) of membrane-filtered (Minisart RC 15, 0.45 μm, Sartorius AG, Göttingen, Germany) samples, after thiolytic degradation, were analyzed using the LC-MS/MS (system 4) in ESI^−^ mode on a RP C18 column (Kinetex, 100 mm × 2.1 mm, 100 Å, 1.7 μm, Phenomenex, Aschaffenburg, Germany). The following gradient consisting of aqueous formic acid (1%) as eluent A and acetonitrile (containing 1% formic acid) as eluent B was used with a flow rate of 0.4 mL/min: isocratic elution at 5% B for 1 min, increasing the content of B to 20% within 5 min, increasing the content of B to 35% within 2 min, then to 100% B within 2 min, isocratic elution at 100% B for 1 min, followed by decreasing the content of B to 5% within 1 min, and finally, holding at 5% B for another 5 min. Using MRM mode, the anthocyanins were measured using the transition reactions in Table S5. The exact concentration of each standard was analyzed by qHNMR, and the analytes were quantified by an external calibration.

#### 2.5.3. Quantitative Analysis of Organic Acids

A IS mixture containing citric acid-^13^C_2_ (0.5 mg/mL), malic acid-*d*_3_ (0.2 mg/mL), tartaric acid-*d*_2_ (2 mg/mL), acetic acid-^13^C_2_ (2 mg/mL), lactic acid-^13^C_2_ (3 mg/mL), oxalic acid-^13^C_2_ (3 mg/mL), and succinic acid-^13^C_2_ (0.6 mg/mL) in acetonitrile/water (50:50, *v*/*v*) was spiked to aliquots (100 µL) of HMW fractions after alkaline hydrolysis. The samples were equilibrated for 1 h, and afterwards, a solution of 3-nitrophenyl hydrazine (3-NPH, 20 μL, 200 mmol/L) in acetonitrile/water (50:50, *v*/*v*) and a solution of *N*-(3-(dimethylamino)propyl)-*N′*-ethylcarbodiimide (EDC, 20 μL, 120 mmol/L) in acetonitrile/water (50:50, *v*/*v*) containing pyridine (6%) were added. The solutions were incubated for 30 min at 40 °C, made up to a volume of 1000 μL with acetonitrile/water (50:50, *v*/*v*), and membrane-filtered (Minisart RC 15, 0.45 μm, Sartorius AG, Göttingen, Germany). Prior to UHPLC-MS/MS measurements (system 3), the samples were diluted 1:100, and an aliquot (1 μL) was injected [39,40]. The sample preparation was performed in triplicates. The quantitation via IS calibration curve and the chromatographic separation was conducted as recently described for citric acid, malic acid, and tartaric acid in red wine [22]. Using the MRM mode, acetic acid, lactic acid, oxalic acid, and succinic acid were analyzed using the transition reactions in Table S5.

Galacturonic acid and (*E*)- and (*Z*)-aconitic acid were quantified in the samples after alkaline hydrolysis without derivatization using a previously published LC-MS/MS approach for red wine [22].

#### 2.5.4. Quantitative Analysis of Polyphenols

According to a recently published LC-MS/MS quantitation approach for polyphenols in red wine [22], the analytes gentisic acid, protocatechuic acid, *p*-hydroxybenzoic acid, *p*-hydroxybenzaldehyde, ferulic acid, phloretic acid, dihydrocaffeic acid, quercetin-3-*O*-*β*-d-glucuronide, methyl gallate, myricetin-3-*O*-glucoside, naringenin, isorhamnetin, quercetin, syringetin, eriodictyol, catechin gallate, epicatechin gallate, epigallocatechin, and gallocatechin were implemented in the method (transition reactions displayed in Table S5). The chromatographic separation by UHPLC-MS/MS (system 2) and the quantitative determination of the target compounds using an external calibration was performed based on the literature [22]. Aliquots (3 µL) of the samples after alkaline hydrolysis and thiolytic degradation, respectively, were membrane-filtered (Minisart RC 15, 0.45 μm, Sartorius AG, Göttingen, Germany) and directly analyzed.

### 2.6. Analysis of Carbohydrates and Alditols by High Performance Ion Chromatography (HPIC)

Carbohydrates and alditols were quantified on a Dionex ICS 5000 ion chromatography system (Dionex, Idstein, Germany) consisting of an ICS 5000 DP-5 dual pump, an ICS 5000 AS-AP autosampler, an ICS 5000 DC-5 detector/chromatography module with column oven, and an ICS 5000 electrochemical detector cell. Aliquots (25 μL) were analyzed on a Carbo Pac MA-1 column (250 mm × 4.0 mm, Thermo Fisher Scientific, Dreieich, Germany) equipped with a guard column (50 mm × 4.0 mm, Thermo Fisher Scientific, Dreieich, Germany) and detected by a pulsed amperometric detector equipped with an Ag/AgCl electrode operating with a standard quadrupole waveform. Analytical separation was conducted at 30 °C at a flow rate of 0.4 mL/min using water and aqueous NaOH (1 M, 90:10) for 140 min. By comparison of retention times and co-chromatography with reference compounds, the identified compounds were further quantified using an external standard calibration. Data were acquired using Chromeleon v7.2 (Dionex, Idstein, Germany).

### 2.7. Isolation and Purification of Chemically Treated HMW Fractions

For NMR interaction studies with treated HMW fractions, defined amounts of each HMW fraction (>50 kDa, 30–50 kDa, and 5–30 kDa) were weighed in for the alkaline hydrolysis (900 mg; 2 h, 80 °C), for the acidic hydrolysis with sulfuric acid (500 mg; 2 h, 100 °C) and hydrochloric acid (500 mg; 24 h, 110 °C), and for the thiolytic degradation (120 mg; 2 h, 60 °C) and treated as described above. After neutralization, the treated HMW fractions were isolated with a Sartoflow Smart system (Sartorius Stedim Biotech, Göttingen, Germany) equipped with a molecular weight cut-off (MWCO) filter membrane (Sartocon^®^ Slice 200 Hydrosart, cut-off 5 kDa, Sartorius Stedim Biotech, Göttingen, Germany). To remove all low-molecular-weight chemical degradation products, the HMW fraction was flushed with water (3.5 L). Afterwards, the treated HMW fractions were lyophilized, and the yields of all isolated fractions were determined gravimetrically.

### 2.8. Nuclear Magnetic Resonance Spectroscopy (NMR)

A Bruker Avance III spectrometer (Bruker, Rheinstetten, Germany) operating at a frequency of 400.13 MHz and equipped with a BBI probe was used for quantitative ^1^H NMR interaction experiments. ^1^H NMR experiments for the identification of new chemical degradation products were conducted on a Bruker Avance Neo 500 MHz system (Bruker, Rheinstetten, Germany) equipped with a cryo-TCI probe. All samples were prepared in 5 × 178 mm NMR tubes (Z107374 USC tubes, Bruker, Faellanden, Switzerland) at 298 °K. Data were acquired and processed using TopSpin 3.6.0 and 4.0.7 (Bruker, Rheinstetten, Germany) and MestReNova 11.0.4 (Mestrelab Research, La Coruña, Spain).

The ^1^H NMR measurements were acquired using a standard 1D pulse sequence (*zg*) from the Bruker software library (TopSpin 3.6.0 and 4.0.7). The probe was locked, tuned, matched, and shimmed with the sample in place. The specific settings for each sample, and the processing of the spectra were performed as described recently [21,41]. An in-house taste compound data base was used for the identification of degradation products in the LMW fractions of chemically treated red wine polymers by means of ^1^H NMR spectroscopy [42].

#### 2.8.1. Quantitative ^1^H NMR Spectroscopy (qHNMR)

Quantitation of the analytes was conducted applying the ERETIC 2 procedure based on the PULCON methodology, following a literature protocol [41]. l-tyrosine (4.34 mmol/L, D_2_O/DCl) was used as an external reference to calibrate the spectrometer. Therefore, the specific proton resonance signal at 7.10 ppm (m, 2H) was used for integration.

#### 2.8.2. Sample Preparation and NMR Interaction Studies

Reference compound solutions of the odorants 3-methylbutanol, 3-methylbutanoic acid, *cis*-whisky lactone, and 3-isobutyl-2-methoxypyrazine were prepared in ethanol-*d*_6_/D_2_O (14/86, *v*/*v*), and their exact concentration was determined by qHNMR. NMR buffer solution (pH 3.8) [21] was added to all solutions before NMR measurements to achieve authentic red wine conditions (ethanol-*d*_6_/D_2_O, 14/86, *v*/*v*; pH 3.8). For NMR-based interaction studies, 60 μL of NMR buffer, 100 μL of HMW fraction, and the respective odorant reference solution were directly mixed in the NMR tube. Ethanol-*d*_6_/D_2_O (14/86, *v*/*v*) was added to reach a total volume of 600 μL and an odorant concentration of 5 mmol/L for every sample. Control solutions without the HMW fraction were prepared in the same manner. The native and chemically treated HMW fractions were used in a concentration of 2.89 g/L. After 30 min of incubation, ^1^H NMR spectra of samples before and after the addition of the HMW fraction were analyzed regarding differences in free odorant concentration, signal shape and intensity, chemical shift, and full width at half-maximum (FWHM). Additionally, the amount of free odorant in the solutions with and without HMW material was monitored by quantitative NMR experiments after several incubation time steps (5 min to 96 h).

### 2.9. Sensory Analyses

#### 2.9.1. General Conditions and Sensory Panel Training

Sensory experiments were conducted with twelve panelists (seven women and five men; age 23–59 years) from the Chair of Food Chemistry and Molecular Sensory Science (Freising, Germany) and from the Leibniz Institute for Food Systems Biology (Freising, Germany) who had given informed consent to participate in the present sensory studies. All assessors had no history of known anosmia and were trained in weekly sensory tests to familiarize themselves with the sensory procedure and the odor of aqueous reference solutions. All samples (10 mL) were prepared in an aqueous solution of ethanol (14%, *v*/*v*, pH 3.8) in glass beakers (total volume 45 mL). Optical differences between the samples were obviated by red lighting. Sensory analyses were conducted in an air-conditioned sensory panel room equipped with single cabins at RT.

#### 2.9.2. Three-Alternative Forced Choice Tests (3-AFC)

Three-alternative forced choice tests were performed to evaluate the influence of the chemical treatment of the polymers on the aroma perception. Therefore, three samples were presented to the assessors. All solutions contained the recently developed aroma reconstitution model of Primitivo red wine [21] in an aqueous solution of ethanol (14%, *v*/*v*, pH 3.8). Additionally, one of the three samples consisted of the aroma reconstitution model and the HMW fraction. The HMW fractions were used before and after chemical degradation in the concentration of 2.89 g/L. Prior to sensory analysis, all solutions were equilibrated for 30 min at RT. The panelists were asked to identify the sample containing the HMW fraction. The significance of the results was evaluated based on the literature [43].

## 3. Results and Discussion

In order to study the correlation of the composition of the HMW fractions and their interaction behavior with different aroma compounds, the previously isolated individual polymer fractions [21] (HMW fractions >50 kDa, 30–50 kDa, and 5–30 kDa) were first chemically decomposed using different analytical approaches.

### 3.1. Identification and Quantitation of Chemical Degradation Products in Red Wine Polymers

Although the composition of red wine polymers was already analyzed in the literature using HPLC-UV/vis, HPIC, HPLC-ESI-FT-ICR-MS, and LC-MS/MS, only 50% of the polymer’s complex structure have been characterized so far [23,24]. To identify new chemical degradation products, new approaches for the analysis of the chemically treated HMW fractions, such as ^1^H NMR experiments and untargeted UHPLC-ToF-MS measurements, were performed. Further, the amount of the degradation compounds was determined for different reaction conditions via HPIC and LC-MS/MS experiments.

For the NMR experiments, the HMW solutions after different chemical treatments (acidic hydrolysis with sulfuric acid or hydrochloric acid, alkaline hydrolysis, and thiolytic depolymerization) were separated in a chemically degraded HMW (>5 kDa) and LMW (<5 kDa) fraction using a crossflow ultrafiltration system. Afterwards, the LMW fraction was measured by ^1^H NMR spectroscopy (Figure S1), and our recently published in-house database [42] was used to identify LMW compounds after chemical degradation of the polymers. For the targeted and untargeted LC-MS measurements, different reaction conditions of each chemical treatment were analyzed regarding differences in the composition by comparing the specific parameters (*m*/*z*, retention time, fragmentation) of the individual compounds with reference compounds. Further, the treated samples were compared to the corresponding untreated ones to ensure that all analyzed compounds were released during the chemical degradation and not already present in the native HMW fractions.

#### 3.1.1. Acidic Hydrolysis with Sulfuric Acid

**Figure 1:**
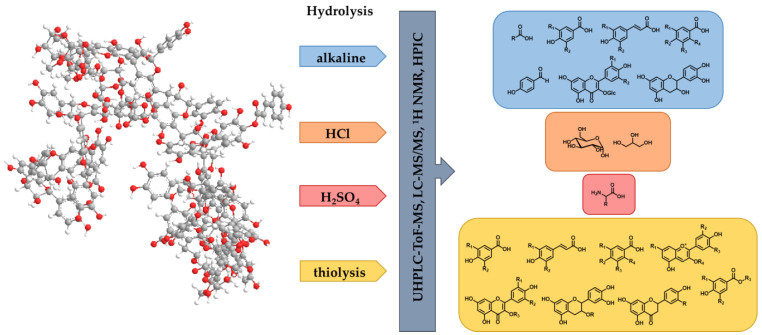
Analyzed compound classes after different chemical treatments of HMW polymers in red wine. The 3D polymer structure is based on the investigations of Wollmann et al., (2013) [23].

After the acidic hydrolysis with sulfuric acid, carbohydrate moieties were detected in the NMR spectra of the LMW fraction (Figure S1). In particular, the anomeric protons exhibited chemical shifts of 4.4–5.5 ppm, and the signals (3.2–4.4 ppm) with typical chemical shifts for the protons attached to the carbon atoms C(2)–C(6) showed the characteristic signal pattern of carbohydrates. Using the in-house database, *α*-arabinose (5.27 ppm), *β*-arabinose (4.55 ppm), *α*-galactose (5.30 ppm), *β*-galactose (4.62 ppm), *α*-mannose (5.21 ppm), *β*-mannose (4.94 ppm), *α*-rhamnose (5.12 ppm), and *β*-rhamnose (4.87 ppm), which were already described in red wine polymers in the literature [23], could be identified. Additionally, the characteristic doublets at 6.96 ppm and 6.51 ppm of the Maillard reaction product 5-hydroxymethylfurfural (HMF) were detected with low intensity, probably as a side-reaction product that was generated through the acid-catalyzed degradation of the carbohydrates [44]. Based on the results of the NMR analysis, the identified carbohydrates were quantified by using HPIC. Additionally, by comparison of the retention times with reference compounds as well as co-chromatography, the compounds glucose, mannitol, glycerol, sorbitol, and trehalose were detected in the hydrolysates using HPIC (Figure 1).

To study the influence of reaction parameters on the chemical degradation of red wine polymers, the quantitative amount of the identified carbohydrates was determined for different reaction times (2 h, 100 °C; 8 h, 100 °C) in the HMW fractions >50 kDa, 30–50 kDa, and 5–30 kDa (Table S6). The prolonged incubation of the polymers under acidic treatment for 8 h, 100 °C, revealed a higher release of carbohydrates in all HMW fractions. While for the degradation at 2 h, 100 °C, all HMW fractions contained a similar amount of carbohydrates (70.3–79.17 µg/mg HMW), HMW fraction >50 kDa exhibited the highest amount for the degradation at 8 h, 100 °C, with 157.92 µg/mg HMW, followed by fractions 30–50 kDa (146.97 µg/mg HMW) and 5–30 kDa (112.84 µg/mg HMW). In all fractions, galactose and mannose were present in the highest concentrations, followed by arabinose, rhamnose, and glucose. Only HMW fraction 5–30 kDa revealed a higher amount of glucose and rhamnose than of mannose after the incubation for 8 h, 100 °C. The compounds mannitol, glycerol, sorbitol, and trehalose were quantified in lower concentrations (0.05–2.05 µg/mg HMW) in all polymeric fractions.

#### 3.1.2. Acidic Hydrolysis with Hydrochloric Acid

Using NMR spectroscopy and UHPLC-ToF-MS, no compounds could be identified by comparison with reference substances in the LMW fraction after hydrochloric acid degradation (Figure S1). As it was already described for red wine polymers [23,24], the reaction conditions were typically used for the detection of amino acids. Therefore, 14 amino acids were identified and quantified in the red wine polymer hydrolysates via a targeted HPLC-MS/MS approach using stable isotope dilution analysis [38] (Figure 1, Table S6). The total content of amino acids was similar for the HMW fractions >50 kDa and 5–30 kDa, with overall concentrations of 10.23 µg/mg HMW and 10.27 µg/mg HMW, respectively. The amount of amino acids in HMW fraction 30–50 kDa was slightly lower, with 8.27 µg/mg HMW. Out of the identified amino acids, aspartic acid was present in the highest concentration in all HMW fractions, followed by serine and glutamic acid in HMW fractions >50 kDa and 30–50 kDa, and glutamic acid and glycine in HMW fraction 5–30 kDa. The lowest amino acid concentration was detected for leucine, isoleucine, phenylalanine, and histidine in all polymeric fractions.

#### 3.1.3. Alkaline Hydrolysis

The ^1^H NMR spectrum of the HMW polymers after alkaline treatment with sodium hydroxide showed signal groups in the entire chemical shift range of 1–8 ppm—in detail, signal groups with high intensity between 3.5 ppm and 4.5 ppm and signal groups with lower intensity in the aliphatic and aromatic region (Figure S1). By comparison with the in-house taste database [42], the methyl group of lactic acid (1.34 ppm) could be identified. The signal groups of additional organic acids such as malic, tartaric, citric, and succinic acid overlapped with the signal groups of other chemical degradation products and were not able to be characterized by NMR experiments. In consequence, organic acids were identified by a targeted LC-MS/MS approach (Table S6). The following organic acids were detected in the HMW hydrolysates: acetic acid, (*E*)-aconitic acid, (*Z*)-aconitic acid, citric acid, galacturonic acid, lactic acid, malic acid, oxalic acid, succinic acid, and tartaric acid. Besides the characteristic signal groups of organic acids, the ^1^H NMR spectrum indicated aromatic compounds to be present in the samples (Figure S1). As described in the literature [23,24], phenolic acids and flavan-3-ols were also found in the alkaline hydrolysates of red wine polymers and show characteristic signal groups in the aromatic region. As no distinct aromatic compounds could be identified by NMR studies, the hydrolysates were analyzed by untargeted UHPLC-ToF-MS experiments in ESI^−^ mode to identify further chemical degradation products (Table S7). By comparison, of the *m*/*z* ratio and the retention time of the compounds in the samples and the reference substances, eight aromatic compounds were identified: caffeic acid, *p*-coumaric acid, dihydrocaffeic acid, gallic acid, phloretic acid, *p*-hydroxybenzoic acid, *p*-hydroxybenzaldehyde, and vanillic acid. Additionally, six literature-known [23,24] aromatic compounds and flavan-3-ols (syringic acid, gentisic acid, protocatechuic acid, ferulic acid, (+)-catechin and (−)-epicatechin), as well as three flavonol glycosides (quercetin-3-*O*-*β*-d-glucuronide, syringetin-3-*O*-*β*-d-glucoside and quercetin-3-*O*-*β*-d-galactoside), were identified by targeted LC-MS/MS measurements (Figure 1).

For the quantitation of the compounds, the alkaline hydrolysis was conducted at 40 °C, 60 °C, and 80 °C to study the effect of the reaction parameters on the degradation of the polymers or on the release of low-molecular-weight components, respectively. The highest amount of organic acids and aromatic compounds was detected for the incubation temperature of 80 °C, followed by 60 °C and 40 °C (Table S6). As already described for the analysis of carbohydrates, incubation at higher temperatures or prolonged incubation times produced higher concentrations of chemical degradation products. The comparison of the different HMW fractions revealed that HMW fraction 5–30 kDa contained the highest amount of organic acids (32.01–69.32 µg/mg HMW), followed by HMW >50 kDa (24.72–52.44 µg/mg HMW) and 30–50 kDa (17.78–45.99 µg/mg HMW) for all used reaction temperatures. Lactic acid, malic acid, and acetic acid were present in high concentrations in all fractions, whereas citric acid, oxalic acid, galacturonic acid, (*E*)-aconitic acid, and (*Z*)-aconitic acid showed only low concentrations in the HMW hydrolysates. Out of all high-molecular-weight fractions, the HMW fraction 30–50 kDa contained the highest amount of releasable aromatic compounds (48.53 and 33.42 µg/mg HMW), followed by HMW fractions 5–30 kDa (38.66 and 29.86 µg/mg HMW) and >50 kDa (31.58 and 26.30 µg/mg HMW) for the reaction temperatures 80 °C and 60 °C; whereas HMW fraction 5–30 kDa showed the highest amount with 31.59 µg/mg HMW for the reaction temperature 40 °C, followed by HMW fractions 30–50 kDa with 28.04 µg/mg HMW and >50 kDa with 19.21 µg/mg HMW. In all analyzed HMW samples, the phenolic acids vanillic acid, syringic acid, *p*-coumaric acid, and protocatechuic acid were present in high concentrations. The remaining phenolic acids, flavan-3-ols, and flavonol glycosides were found in traces.

#### 3.1.4. Thiolytic Depolymerization

After thiolytic degradation of the red wine polymers, the NMR spectrum of the LMW fraction mainly showed signals in the range of 3–8 ppm (Figure S1). Compared to the spectrum after alkaline hydrolysis, no signals in the aliphatic region and fewer signals in the range of 3.5–4.5 ppm could be detected, whereas most of the signals were located in the aromatic region. According to the literature [29,45,46], benzyl mercaptan is used as a nucleophilic degradation agent to decompose the complex structure of tannins and proanthocyanidins in plant material. So far, mainly flavan-3-ols and their galloylated derivates, flavan-3-ol-thioethers, anthocyanins, pyranoanthocyanins, and acylated anthocyanins, were detected after thiolysis of the red wine polymers [23,24]. In this study, untargeted UHPLC-ToF-MS measurements in ESI^−^ and ESI^+^ mode were selected to identify the single degradation products after thiolysis in the HMW fractions (Tables S8 and S9).

In ESI^−^ mode, the flavan-3-ols catechin and epicatechin and their galloylated derivates catechin gallate, epicatechin gallate, epigallocatechin, and gallocatechin, as well as the flavonol quercetin, were identified. Besides these literature-known compounds [24], the following analytes could be identified in red wine polymers for the first time: eriodictyol, methyl gallate, isorhamnetin, naringenin, quercetin-3-*O*-*β*-d-galactoside, and syringetin (Table S8). Using a database of literature-known compounds in red wine [1,2,3,23,24,31,32,33,34,35,36,37], the flavan-3-ol-thioethers were also identified by untargeted measurements. As these degradation products could not be identified by reference compounds, and as their formation during the thiolysis of red wine polymers was discussed in detail in the literature [23], the flavan-3-ol-thioethers were not analyzed further in this study. The identified compounds were quantitively determined via a targeted LC-MS/MS approach using external calibration (Table S6). In total, twelve phenolic compounds were investigated in the HMW samples after thiolysis by these targeted experiments (Figure 1). Three different reaction parameters, which were already described in the literature [23,30], were set for the thiolytic degradation (2 h 40 °C, 2 h 60 °C, and 2 min 90 °C) to study the influence of reaction time and temperature on the release of low-molecular-weight compounds. The total amount of the phenolic compounds was very similar for all reaction conditions and HMW fractions. In detail, all phenolic compounds were found in traces in the HMW hydrolysates. The highest concentration of flavan-3-ols was present after incubation at 2 min 90 °C, followed by 2 h 40 °C and 2 h 60 °C. Compared to the other used degradation methods, no clear trend was detectable. Suo et al., (2019) [24] demonstrated that depending on the thiolysis reaction conditions, by-products and incomplete reactions can occur. However, HMW fraction 5–30 kDa contained the highest amount of flavan-3-ols and the lowest amount of phenolic compounds for all reaction conditions. Among the flavan-3-ols and their derivates, (−)-epicatechin and (+)-catechin were present in higher concentrations than the galloylated forms.

Using ESI^+^ mode in the UHPLC-ToF-MS experiments, six anthocyanins and anthocyanidins, respectively, could be detected by comparison with reference substances (Figure 1, Table S9). Further, in total, five anthocyanins and six anthocyanidins were quantified in the thiolytic treated solutions via targeted LC-MS/MS experiments (Table S6). HMW fraction 5–30 kDa revealed the highest amount of this structural class for all reaction parameters, followed by HMW 30–50 kDa and >50 kDa. In all fractions, malvidin and malvidin-3-*O*-glucoside were present in the highest concentrations. In general, anthocyanidins were detected in slightly higher amounts than anthocyanins. Comparing the reaction conditions showed a higher concentration of anthocyanins and anthocyanidins for 2 h 60 °C than for 2 min 90 °C and 2 h 40 °C, demonstrating no clear trend of the reaction parameters again. In comparison to the literature data of anthocyanidins and their derivates in red wine polymers [24], no pyranoanthocyanins and acylated anthocyanins were analyzed due to the lack of reference substances. However, these compounds may also play a role in the composition of the HMW fractions in red wine.

#### 3.1.5. Summary

In summary, the following structural classes of chemical degradation products could be detected in the HMW fractions: carbohydrates and polyols after acidic hydrolysis with sulfuric acid; amino acids after acidic hydrolysis with hydrochloric acid; organic acids, phenolic acids, flavonol glycosides, and flavan-3-ols after alkaline hydrolysis; and flavan-3-ols, galloylated flavan-3-ols, flavonols, phenolic compounds, anthocyanins, and anthocyanidins after thiolysis. For the first time, the following compounds could be identified in red wine polymers: rhamnose, mannitol, sorbitol, trehalose, pyroglutamic acid, *p*-coumaric acid ethyl ester, acetic acid, oxalic acid, (*E*)-aconitic acid, (*Z*)-aconitic acid, *p*-hydroxybenzaldehyde, phloretic acid, dihydrocaffeic acid, quercetin-3-*O*-*β*-d-glucuronide, syringetin-3-*O*-*β*-d-glucoside and quercetin-3-*O*-*β*-d-galactoside, gallic acid ethyl ester, *trans*-caffeic acid ethyl ester, methyl gallate, myricetin-3-*O*-glucoside, naringenin, isorhamnetin, syringetin, and eriodictyol.

Except for the thiolytic depolymerization, harsher reaction conditions caused a higher amount of degradation products, but a similar composition of the individual compounds. In total, by calculating the sum of all constituents in the single fractions, 62% of the HMW fraction >5 kDa could be analyzed. In detail, HMW fraction >5 kDa consisted of 23% carbohydrates, 17% organic acids, 12% phenolic compounds, 5.7% anthocyanins and anthocyanidins, 2.9% amino acids, and 1.6% flavan-3-ols. Compared to literature data of HMW fraction >5 kDa isolated from a Bordeaux red wine using gel absorption chromatography [23], the compositional analysis of Primitivo HMW >5 kDa showed a similar trend in the constitution of the polymers. Carbohydrates were present in the highest concentration in both polymers, whereas flavan-3-ols exhibited lower concentrations in the Primitivo polymer. This difference might occur due to the different grape varieties or production processes.

All Primitivo HMW fractions showed similar degradation behaviors with slight differences in their constitution. The highest amount of degradation products was mostly detected in HMW fraction 5–30 kDa, followed by HMW fraction 30–50 kDa and >50 kDa (Table S6). To study the correlation between the structural composition of the HMW fractions and their interaction affinities with odorants, NMR experiments with chemically degraded polymers were performed.

### 3.2. Isolation of HMW Fractions after Chemical Degradation

For the isolation of chemically degraded polymers, the single HMW fractions were treated using one reaction condition of each chemical treatment (alkaline hydrolysis, 2 h, 80 °C; acidic hydrolysis with sulfuric acid, 2 h, 100 °C; acidic hydrolysis with hydrochloric acid, 24 h, 110 °C; thiolysis, 2 h, 60 °C). Furthermore, the treated solutions were separated in a HMW (>5 kDa) and an LMW (<5 kDa) fraction, and the salt generated during the neutralization of the reactions was removed via a second ultrafiltration step. Therefore, the HMW fraction was flushed with water until no more distinct resonance signals, representing low-molecular-weight material, were present in the ^1^H NMR spectrum. After isolation, the chemically degraded HMW fractions were lyophilized, and their yields were determined gravimetrically (Table 1). Therefore, the quotient of the yield of the HMW fraction after chemical treatment and the used amount of native HMW fraction was determined. For all fractions, the lowest proportion was observed after acidic hydrolysis with sulfuric acid and hydrochloric acid, respectively. Besides the degradation of carbohydrates and amino acids, the acidic conditions also cause an oxidation of the flavonoids, clearly visible as a black, not soluble residue. In detail, flavan-3-ols such as epicatechin can form an acid catalyzed benzyl cation that can react with other flavan-3-ols and form polymeric structures [47]. The black residue was removed before ultrafiltration and could not be analyzed via the analytical approaches used to identify chemical degradation products. Nevertheless, this side reaction must be considered when evaluating the correlation between the chemical composition of red wine polymers and their interaction behavior in the NMR experiments. The highest proportion of polymers after chemical degradation in relation to the amount of native HMW fraction of each individual fraction was determined after thiolysis for HMW fractions 30–50 kDa (73%) and 5–30 kDa (59%), whereas HMW fraction >50 kDa exhibited the highest amount of treated polymer after alkaline hydrolysis (72%). With exception of HMW fraction 5–30 kDa, the treated HMW fractions showed similar proportions of 59–73% after the alkaline hydrolysis and thiolytic depolymerization. In general, HMW fraction 5–30 kDa exhibited the lowest amount after all chemical degradations, showing a correlation with the highest amount of identified and quantified chemical degradation products.

### 3.3. NMR-Based Interaction Studies between Chemically Degraded Polymers and Odorants

**Figure 2:**
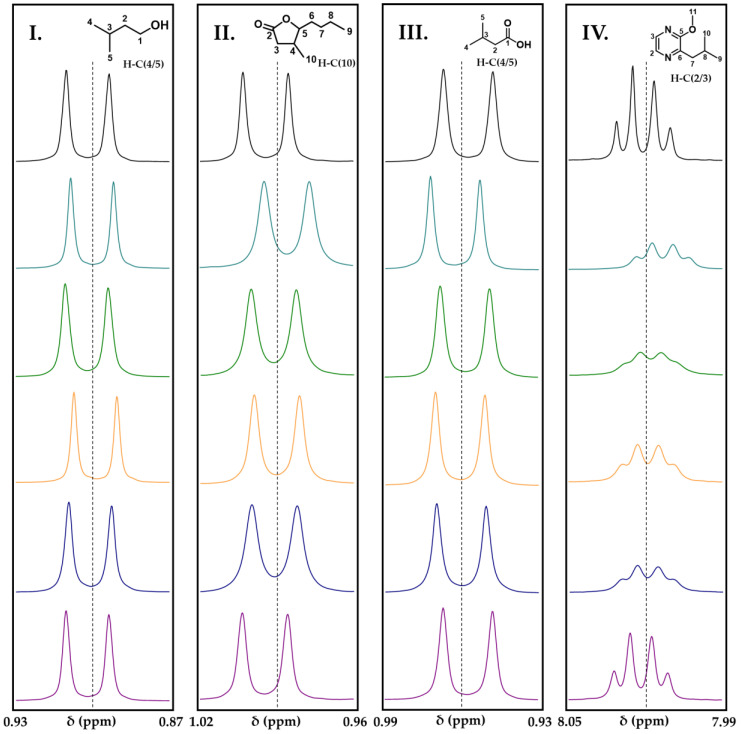
Excerpts of qHNMR spectra (400 MHz; ethanol-*d*_6_/D_2_O, 14/86, *v*/*v*; pH 3.8; 298 K) of NMR-based interaction studies of 3-methyl-1-butanol (**I**), *cis*-whisky lactone (**II**), 3-methylbutanoic acid (**III**), and 3-isobutyl-2-methoxypyrazine (**IV**) with HMW fractions after chemical degradation of HMW fraction 30–50 kDa (control solution without HMW (black), native HMW (light blue), HMW after alkaline hydrolysis (green), HMW after acidic hydrolysis with H_2_SO_4_ (orange), HMW after thiolytic depolymerization (dark blue), and HMW after acidic hydrolysis with HCl (purple)) in equal concentration (2.89 g/L) after 30 min of incubation at RT.

**Figure 3:**
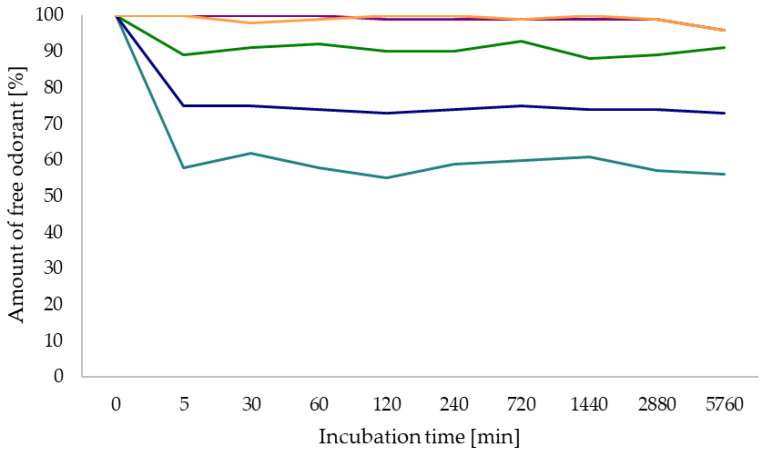
Percentage of the free 3-isobutyl-2-methoxypyrazine in the solution after various incubation times (5 min–96 h) with the HMW 30–50 kDa fraction (2.89 g/L) after different chemical degradations (light blue: native polymer; green: polymer after alkaline hydrolysis; orange: polymer after acidic hydrolysis with H_2_SO_4_; purple: polymer after acidic hydrolysis with HCl; dark blue: polymer after thiolytic depolymerization). Values were calculated in relation to the control solution without added HMW fraction. According to the validation of the qHNMR approach in the literature [41], the precision of the method was found to be ≤2%.

In order to analyze the correlation of the observed phenomenon of odorant polymer interactions as previously described in the literature [21], the molecular structures of the odorants, and the composition of the HMW fractions, NMR interaction studies with chemically degraded polymers were performed. Therefore, four key aroma compounds in red wine (3-methylbutanol, *cis*-whisky lactone, 3-methylbutanoic acid, and 3-isobutyl-2-methoxypyrazine) were incubated with the HMW fractions, each compound representing one interaction scenario of odorant polymer interactions [21]. To study the effect of the chemical degradation on the interactions with these compounds, the NMR interaction studies with treated polymers were compared to the ones with the non-treated polymer. Therefore, the fraction with the highest interaction potential, HMW fraction 30–50 kDa, was chemically treated as described above (thiolysis, acidic, alkaline hydrolysis), and all treated fractions were spiked to the odorants in the same concentration as the original untreated ones (Figure 1). The ^1^H NMR spectra of the odorant with and without added polymers were qualitatively evaluated after 30 min of incubation at RT regarding differences in signal shape, chemical shift, signal intensity, and full width at half-maximum (FWHM) (Figure 2). Additionally, the recently described quantitative aroma binding effect of pyrazines [21], which resulted in a lower concentration of the free odorant after the addition of the HMW fractions, was analyzed for the HMW fractions after chemical treatment. Therefore, 3-isobutyl-2-methoxypyrazine was incubated with the native HMW fraction 30–50 kDa and the corresponding treated HMW fractions in equal concentration at different time steps (5 min to 96 h), and the amount of free odorant was determined via qHNMR [41] (Figure 3).

As already described, chemical shift changes of <1 Hz were considered as no interaction as they did not show any perceivable effect in the human sensory experiments [21]. For instance, NMR interaction studies demonstrated that 3-methylbutanol did not interact with the HMW fraction, independent of the chemical treatment, since each addition of the HMW material did not show chemical shift changes >1 Hz. The other analyzed odorants revealed reduced interactions after the addition of the treated HMW fraction 30–50 kDa compared to the untreated, native one. In detail, *cis*-whisky lactone showed no interactions with the polymer after acidic hydrolysis with hydrochloric acid and a reduced shielding-effect for the HMW fraction after sulfuric acid treatment (1.75 Hz), thiolysis (1.41 Hz), and alkaline hydrolysis (1.22 Hz), compared to the native HMW fraction (3.48 Hz). The odorant 3-methylbutanoic acid revealed a reduced interaction affinity after incubation with polymers after acidic hydrolysis with sulfuric acid, and no more interactions after all the other chemical treatments. For 3-isobutyl-2-methoxypyrazine, the NMR spectrum showed a chemical shift change of 3.62 Hz to lower frequencies after incubation with the native polymer. In comparison, the HMW fraction after alkaline hydrolysis caused a difference of 1.32 Hz, whereas after the addition of the HMW fractions of the other chemical treatments, the signal group barely showed any chemical shift changes. Additionally, the signal shape and line broadening of H-C (2/3) of the pyrazine after incubation with the alkaline hydrolyzed polymer was similar to the native one, whereas the signal spiked with the polymer after acidic hydrolysis with hydrochloric acid demonstrated a clear signal shape and no line broadening compared to the reference compound (Figure 2).

The quantitative measurements of the pyrazine and different chemically degraded polymers revealed a higher concentration of the free odorant compared to the native polymer, which caused an immediate decline in concentration of the free 3-isobutyl-2-methoxypyrazine to 56% [21]. In detail, the polymer after acidic hydrolysis with hydrochloric and sulfuric acid showed a decline in the concentration of the free odorant to 96%, followed by the polymer after alkaline hydrolysis (91%) and thiolysis (73%). While the HMW fraction after thiolytic and alkaline degradation still showed quantitative interactions with the aroma compound, the HMW fractions after acidic hydrolysis barely showed any quantitative effect. For this reason, the effects on the quantitative aroma binding effect were similar to the ones on the qualitative changes in the NMR spectra after the addition of treated HMW fractions (Figure 3).

These observations clearly indicate that the chemical degradation products must play a role in the formation of interactions of the polymer with the odorants, as previously shown for NMR studies with alkaline-treated coffee melanoidins [48]. Further, a correlation between the reduction of the interactions in the NMR experiments and the importance of the chemical degradation products for the interactions could be assumed. In red wine, the polymer after acidic hydrolysis with sulfuric acid revealed a lack of carbohydrates and, additionally, a lack of polymerized phenolic structures. In addition, after acidic hydrolysis with hydrochloric acid, the HMW fraction missed the polymerized phenolic structures as well as amino acids. After alkaline hydrolysis, organic acids, phenolic acids, flavonol glycosides, and flavan-3-ols were no longer available as aroma interaction sites; whereas after thiolysis, parts of the polymer structure were degraded to flavan-3-ols, galloylated flavan-3-ols, flavonols, phenolic compounds, anthocyanins, and anthocyanidins. Besides the lack of degradation products, new interaction sites at the polymer could occur, which led to a different interaction behavior.

Based on the chemical composition of the HMW fraction, amino acids and polymeric phenols had the highest impact on the interactions with *cis*-whisky lactone, followed by the chemical degradation products after alkaline hydrolysis, thiolysis, and acidic hydrolysis with sulfuric acid. Nevertheless, all degraded polymers demonstrated an impact on the interactions with similar intensities. For 3-methylbutanoic acid, all chemical degradation products played a role in this odorant polymer interaction type, whereby the carbohydrates and oxidized polyphenols caused the lowest impact on the interactions. Compared to the chemical composition of the different polymers, the interaction sites for the π–π interactions of 3-isobutyl-2-methoxypyrazine were mainly polymerized phenolic structures and the chemical degradation products of alkaline hydrolysis and thiolysis. Additionally, also carbohydrates and amino acids indicated to play a role in the formation of interactions with aliphatic structural features of the odorant.

The comparison of DFT studies in the literature [25] with the NMR interaction studies with chemically degraded polymers showed that the intensity of the interactions was highly dependent on the structural properties of the odorants and polymer segments. In detail, the DFT calculations have identified (+)-catechin as the polymer segment within the selected set that exhibits the highest binding affinity for aroma compounds [25]. This compound was also found in the LMW fractions after all chemical treatments and could be the main interaction site in red wine polymers. Nevertheless, every odorant showed different trends for the interaction with chemically degraded polymers. For 3-methylbutanol, no clear trend in the NMR experiments could be observed as there were no interactions detectable. The odorant *cis*-whisky lactone showed the lowest interaction activity with the polymer after acidic hydrolysis with hydrochloric acid, clearly indicating effects of amino acids and polymerized phenolic substances. In contrast, the interaction affinity of the polymer after acidic hydrolysis with sulfuric acid was reduced compared to the native polymer but more intense than for the other chemical degradations. These observations could be confirmed by DFT experiments, as the lactone was mainly interacting with (+)-catechin, *p*-coumaric acid, and aspartic acid but interacting less with carbohydrates [25]. For 3-methylbutanoic acid, only a small binding affinity with all polymer segments was observed in the simulation experiments, and there was no detectable effect in the NMR experiments and sensory studies of 1:1 complexes of single-polymer segments and the odorant [25]. In the present study, all degradations led to a reduced interaction activity, indicating that the small interaction activities of all chemical degradation products must be responsible for the de-shielding effect with the native polymer. According to literature, the aroma compound 3-isobutyl-2-methoxypyrazine formed very stable π–π complexes with (+)-catechin [25]. As (+)-catechin was present after all chemical degradations in the LMW, a correlation between the amount of the released flavan-3-ol after chemical hydrolysis and the reduced interaction activity of the treated polymer could be detected. As the yields of the treated HMW fractions showed (Table 1), a high amount of the fractions was lost after acidic hydrolysis with sulfuric acid and hydrochloric acid due to polymerization of phenolic structures. As these chemical treatments caused the highest release of flavan-3-ols from the polymers, the lowest interaction activity could be observed for the polymers after acidic hydrolysis. After thiolysis, more flavan-3-ols were quantified than after alkaline hydrolysis. This effect could also be detected in the NMR interaction studies with treated polymers. Compared to the acidic hydrolysis, after alkaline hydrolysis and thiolysis, a loss of signal shape and an effect of the FWHM was still monitored. This observation could be explained by the high interaction affinity of the pyrazine, especially through π–π stacking with aromatic systems. After alkaline hydrolysis and thiolysis, new interaction sites in the polymer could occur that still cause interactions with the odorant, whereas after the acidic hydrolysis, no more interaction sites were available.

In conclusion, the NMR interaction studies with the polymers after chemical treatment were well in line with the recently published DFT-based approach [25] and could give an insight into the interacting segments of the red wine polymer. Mainly flavan-3-ols were identified as polymer segments with strong interacting affinity, especially for odorants with aromatic systems. Phenolic compound classes were already discussed as important intermolecular interaction sites for aroma binding in food systems in the literature. For instance, one- and two-dimensional NMR studies revealed π–π interactions of phenolic compounds and aromatic odorants, supporting the hypothesis that these low-molecular-weight complexes also occur in the formation of polymer odorant interactions [15,16]. In coffee, phenolic compounds—especially hydroxycinnamic acids—were also identified as the main interaction sites in HMW melanoidins with aromatic compounds such as, e.g., pyrazines [48]. The importance of phenols in the aroma release has been shown in red wine as well. In sensory studies, catechin strongly influenced the orthonasal perception of various esters, e.g., ethyl butanoate, which was attributed to hydrophobic forces and steric effects [19]. Further studies investigated either an increase or a reduction of the perceived intensity of certain aroma compounds after the addition of polyphenols like gallic acid, catechin, or caffeic acid [18,20]. Besides these studies, an altered flavor release of volatile compounds was also shown for phenolic extracts and tannins recently [13,17,49,50,51,52]. At the molecular level, non-covalent interactions such as hydrophobic forces or π–π interactions were suspected [50]. Although all studies showed an influence of the phenolic compounds on the aroma release and perception of red wine, only single compounds were analyzed in model systems. The formation of polymeric structures and their interaction with aroma compounds was not considered and could be described by the results of the NMR interaction studies with chemically degraded polymers and DFT experiments for the first time. Nevertheless, the literature findings support the thesis that polyphenolic compounds are important interaction sites in the red wine polymers.

Additionally, the HMW fractions >50 kDa and 5–30 kDa, which showed reduced odorant polymer interactions compared to HMW fraction 30–50 kDa, were chemically treated, and NMR interaction studies with the four aroma compounds were performed (Figures S2 and S3). Compared to the treated HMW fraction 30–50 kDa, similar trends in the interaction activity were observed, caused by a similar degradation behavior of all fractions. For this reason, the correlation of the chemical degradation and the interaction activity could be confirmed, and the novel approach of interaction studies of treated HMW fractions via NMR spectroscopy could be verified.

### 3.4. Human Sensory Experiments after Chemical Degradation of the Polymers

As recently described in literature, odorant and tastant polymer interactions influenced the human sensory perception of aroma- and taste-active compounds in red wine. In detail, odorants and tastants, which showed changes in the NMR spectrum after the addition of native polymers, also revealed a significant difference in their sensory perception [21,22]. To verify the concept of chemically degraded polymers leading to a reduction of odorant polymer interactions, human sensory experiments were carried out. Therefore, an aroma reconstitution model of Primitivo red wine was prepared and spiked with native and treated HMW fraction >5 kDa. Using three-alternative forced choice tests, the sensory panel was asked to identify the differing sample out of three samples, one sample containing the HMW fraction and two samples without polymers. Due to the strong intrinsic aroma of the HMW fraction >5 kDa after thiolysis, no human sensory experiments could be performed with this fraction. Nevertheless, sensory studies with the native HMW fraction >5 kDa and the HMW fraction >5 kDa after alkaline and acidic hydrolysis were conducted. For the native HMW fraction >5 kDa, the panelists noticed a significant difference in the sensory perception of the aroma recombinant, whereas for the treated high-molecular-weight fractions, no significant differences were observed (Table 2). In conclusion, the chemical treatment influenced the interaction behavior with the aroma compounds, as already shown in the NMR interaction studies.

## 4. Conclusions

In summary, application of untargeted UHPLC-ToF-MS and NMR screenings, as well as targeted LC-MS/MS and HPIC quantitation, led to the identification of degradation products in red wine polymers after different chemical treatments. By applying an NMR-based approach, the correlation of the chemical composition of the polymers and their interaction affinity with odorants could be analyzed. In comparison to DFT studies, flavan-3-ol derivates could be determined as the main interaction sites in red wine polymers, especially for the group of pyrazines, which are able to form π–π complexes with other aromatic systems. Additionally, human sensory studies verified the reduction of odorant polymer interactions by treated HMW fractions, demonstrating the influence of these interactions on the sensory perception of red wine. For the first time, important insights on the formation of interactions between odorants and polymers in red wine could be obtained on a molecular level. Furthermore, through targeted chemical treatment of natural red wine polymers, the formation and the sensory effects of odorant polymer interactions can be easily influenced in the future, e.g., by adding tailor-made polymers.

## Figures and Tables

**Table 1:** Yields of high-molecular-weight fractions isolated after chemical degradation.

Chemical Degradation	Yield of HMW Fraction [%]		
	>50 kDa	30–50 kDa	5–30 kDa
Alkaline Hydrolysis	72	60	23
Acidic Hydrolysis (H_2_SO_4_)	30	24	18
Acidic Hydrolysis (HCl)	11	6	6
Thiolysis	64	73	59

**Table 2:** 3-AFC tests of the Primitivo aroma reconstitution model with native and treated HMW fraction >5 kDa.

HMW Fraction	*p* Value ^a^ (%)	Significance ^b^
Native	0.1	S
Alkaline Hydrolysis	21.2	NS
Acidic Hydrolysis (H_2_SO_4_)	11.1	NS
Acidic Hydrolysis (HCl)	11.1	NS

^a^ *p* values were calculated according to literature [43]. ^b^ NS, not significant (*p* > 5%); S, very highly significant (*p* ≤ 0.1%).

## Data Availability

Data are contained within the article and Supplementary Materials. All data used in this publication are saved at the Chair of Food Chemistry and Molecular Sensory Science, Freising, Germany.

## Supplementary Materials

The following supporting information can be downloaded at https://www.mdpi.com/article/10.3390/foods13040526/s1: Chemicals and Ultra-high-performance liquid chromatography—triple quadrupole mass spectrometry (UHPLC-MS/MS) systems; Table S1: Retention Time, Calibration Curves, and Coefficients of Determination of Quantified Substances using LC-MS/MS; Table S2: Validation Experiments for Quantitation of Chemical Degradation Products after Alkaline Hydrolysis using LC-MS/MS; Table S3: Validation Experiments for Quantitation of Chemical Degradation Products after Thiolytic Depolymerization by means of LC-MS/MS; Table S4: LOD and LOQ of Chemical Degradation Products using LC-MS/MS; Table S5: MRM transitions of analyzed compounds; Table S6: Concentrations of Chemical Degradation Products of Hydrolysates of Red Wine Polymers; Table S7: UHPLC-ToF-MS Data of HMW fraction >5 kDa after alkaline hydrolysis in ESI^-^ mode; Table S8: UHPLC-ToF-MS Data of HMW fraction >5 kDa after thiolytic depolymerization in ESI^-^ mode; Table S9: UHPLC-ToF-MS Data of HMW fraction >5 kDa after thiolytic depolymerization in ESI^+^ mode; Figure S1: Excerpts of ^1^H NMR spectra (1–8 ppm; 500 MHz; ethanol-*d*_6_/D_2_O, 14/86, *v*/*v*; pH 3.8; 298 K) of low-molecular-weight fractions <5 kDa after chemical degradation (A: acidic hydrolysis (H_2_SO_4_) 2 h 100 °C; B: acidic hydrolysis (HCl); C: alkaline hydrolysis 2 h 60 °C; D: thiolytic depolymerization 2 h 60 °C); Figure S2: Excerpts of qHNMR spectra (400 MHz; ethanol-*d*_6_/D_2_O, 14/86, *v*/*v*; pH 3.8; 298 K) of NMR based interaction studies of 3-methyl-1-butanol (I), *cis*-whisky lactone (II), 3-methylbutanoic acid (III) and 3-isobutyl-2-methoxypyrazine (IV) with HMW fractions after chemical degradation of HMW fraction >50 kDa (control solution without HMW (black), native HMW (light blue), HMW after alkaline hydrolysis (green), HMW after acidic hydrolysis with H_2_SO_4_ (orange), HMW after thiolytic depolymerization (dark blue), and HMW after acidic hydrolysis with HCl (purple)) in equal concentration (2.89 g/L) after 30 min of incubation at RT; Figure S3: Excerpts of qHNMR spectra (400 MHz; ethanol-*d*_6_/D_2_O, 14/86, *v*/*v*; pH 3.8; 298 K) of NMR based interaction studies of 3-methyl-1-butanol (I), *cis*-whisky lactone (II), 3-methylbutanoic acid (III) and 3-isobutyl-2-methoxypyrazine (IV) with HMW fractions after chemical degradation of HMW fraction 5–30 kDa (control solution without HMW (black), native HMW (light blue), HMW after alkaline hydrolysis (green), HMW after acidic hydrolysis with H_2_SO_4_ (orange), HMW after thiolytic depolymerization (dark blue), and HMW after acidic hydrolysis with HCl (purple)) in equal concentration (2.89 g/L) after 30 min of incubation at RT.
