# Supplementary material for: Chemical Characterization of Red Wine Polymers and Their Interaction Affinity with Odorants
Source: Foods. 2024 Feb 8;13(4):526. doi: 10.3390/foods13040526 (PMC10888325; doi:10.3390/foods13040526)
Supplement: Supplementary file 1 [file foods-13-00526-s001.zip › foods-2842747-supplementary.pdf]

Supplementary Materials

Chemical Characterization of Red Wine Polymers and Their Interaction Affinity with Odorants

Anna Maria Gabler [1], Annalena Ludwig [1], Florian Biener [1], Magdalena Waldner [1], Corinna Dawid [1,2,*] and Oliver Frank [1,*]

[1] Chair of Food Chemistry and Molecular Sensory Science, TUM School of Life Sciences, Technical University of Munich, Lise-Meitner-Str. 34, D-85354 Freising, Germany; annamaria.gabler@tum.de (A.M.G.); annalenaludwig@gmx.de (A.L.)
[2] Professorship for Functional Phytometabolomics, TUM School of Life Sciences, Technical University of Munich, Lise-Meitner-Str. 34, D-85354 Freising, Germany
* Correspondence: corinna.dawid@tum.de (C.D.); oliver.frank@tum.de (O.F.); Tel.: +49-8161-71-2901 (C.D.); +49-8161-71-2910 (O.F.)

2.2. Chemicals

The following compounds were purchased commercially (sources in parentheses): oxalic acid-$^{13}C_2$ (Biozol, Eching, Germany); protocatechuic acid (Carl Roth GmbH, Karlsruhe, Germany); tartaric acid-*d*$_2$ (CDN Isotopes, Pointe-Claire, Canada); acetic acid-$^{13}C_2$ (Eurisotop, Saarbrücken, Germany); catechin gallate, cyanidin, cyanidin-3-*O*-glucoside, delphinidin, delphinidin-3-*O*-glucoside, dihydrocaffeic acid, epicatechin gallate, epigallocatechin, eriodicytol, gallocatechin, gentisic acid, isorhamnetin, isorhamnetin-3-*O*-glucoside, malvidin, malvidin-3-*O*-glucoside, methyl gallate, myricetin-3-*O*-glucoside, naringenin, pelargonidin, peonidin, peonidin-3-*O*-glucoside, petunidin, petunidin-3-*O*-glucoside, phloretic acid, quercetin, quercetin-3-*O*-*β*-D-galactoside, quercetin-3-*O*-*β*-D-glucuronide, syringetin, syringetin-3-*O*-*β*-D-glucoside (Extrasynthese, Genay, France); arabinose, ferulic acid, gallic acid ethyl ester, succinic acid, vanillic acid (Fluka, Neu-Ulm, Germany); *p*-coumaric acid ethyl ester (Key Organics, Camelford, UK); L-aspartic acid, L-glutamic acid, glycine, L-histidine, hydrochloric acid, *p*-hydroxybenzoic acid, L-isoleucine, L-leucine, L-lysine, mannose, L-phenylalanine, potassium hydrogen phosphate, potassium hydroxide, pyridine, L-pyroglutamic acid, L-serine, sodium hydroxide, sulfuric acid, L-threonine (Merck, Darmstadt, Germany); *trans*-caffeic acid ethyl ester (Phytolab, Vestenbergsgreuth, Germany); acetic acid, (*E*)-aconitic acid, (*Z*)-aconitic acid, L-alanine, L-arginine, ascorbic acid, benzyl mercaptan, caffeic acid, caftaric acid, castalagin, (+)-catechin, citric acid, citric acid-$^{13}C_2$, *p*-coumaric acid, deuterium chloride, deuterium oxide, (−)-epicatechin, ethanol-*d*$_6$, ethylenediaminetetraacetic acid (EDTA), formic acid, furan-2-carboxylic acid, galacturonic acid monohydrate, galactose, gallic acid, glucose, L-glutamine, glycerol, *p*-hydroxybenzaldehyde, 3-isobutyl-2-methoxypyrazine, lactic acid, lactic acid-$^{13}C_2$, malic acid, malic acid-*d*$_3$, mannitol, 3-methyl-1-butanol, 3-methylbutanoic acid, 3-nitrophenylhydrazine hydrochloride (3-NPH), *N*-(3-

(dimethylamino)propyl)-*N′*-ethylcarbodiimide (EDC), oxalic acid, protocatechuic acid ethyl ester, rhamnose, sodium azide, sorbitol, succinic acid-$^{13}C_2$, syringic acid, tartaric acid, trehalose, trimethylsilyl propionic acid (TMSP), *cis*-whisky lactone (Sigma-Aldrich, Steinheim, Germany); ethanol (VWR, Darmstadt, Germany).

2.5. Ultra-High-Performance Liquid Chromatography – Triple Quadrupole Mass Spectrometry (UHPLC-MS/MS)

System 1 was a Dionex Ultimate 3000 LC system (Dionex, Idstein, Germany) consisting of two pumps (HPG-3400SD), a degasser (SRD-3400), an autosampler (WPS-3000TSL, temperature set to 10 °C), and a column oven (TCC-3000SD, temperature set to 50 °C). The LC system was connected to an API 4000 QTrap mass spectrometer (Sciex, Darmstadt, Germany) operating in the positive electrospray ionization mode (ESI^+). The ion spray voltage was set at 5500 V, curtain gas at 35, nebulizer gas at 55, heater gas at 65, and source temperature at 450 °C. Data were acquired using Analyst software (version 1.6.2, AB Sciex, Darmstadt, Germany).

System 2 used a Shimadzu Nexera X2 UHPLC (Shimadzu, Duisburg, Germany) equipped with two pumps (LC-30AD), a degasser (DGU-20A5R), an autosampler (SIL-30AC, temperature set to 10 °C), a column oven (CTO-30A, temperature set to 40 °C), and a controller (CBM-20A), connected to an API QTrap 5500 mass spectrometer (Sciex, Darmstadt, Germany). Detection was achieved using an ESI^- mode with the following source parameters: ion spray voltage, −4500 V; curtain gas, 40; nebulizer gas, 55; heater gas, 65; and source temperature, 450 °C. Data were acquired using Analyst software (version 1.6.2, AB Sciex, Darmstadt, Germany).

System 3 consisted of a Shimadzu Nexera X2 UHPLC (Shimadzu, Duisburg, Germany) equipped with two pumps (LC-30AD), a degasser (DGU-20A5R), an autosampler (SIL-30AC, temperature set to 15 °C), a column oven (CTO-30A, temperature set to 40 °C), and a controller (CBM-20A). The UHPLC system was connected to a QTRAP 6500 mass spectrometer (AB Sciex, Darmstadt, Germany) operating in the negative electrospray ionization mode (ESI^-) and controlled by the Analyst software (version 1.6.3, AB Sciex, Darmstadt, Germany). Detection was achieved using the following source parameters: ion spray voltage, −4500 V; curtain gas, 35 psi; nebulizer gas, 55 psi; heater gas, 65 psi; and source temperature, 450 °C.

System 4 comprised an ExionLC (Sciex, Darmstadt, Germany) equipped with two LC pump systems (ExionLC AD Pump), an ExionLC degasser, an ExionLC AD autosampler (temperature set to 10 °C), an ExionLC AC column oven (temperature set to 40 °C), and an ExionLC controller, coupled with a QTrap 6500+ mass spectrometer (Sciex, Darmstadt, Germany). The ion spray voltage was set at −4500 V in the ESI^- mode, curtain gas at 40, nebulizer gas at 55, heater gas at 65, and source temperature at 450 °C. Data were acquired using Analyst software (version 1.6.3, AB Sciex, Darmstadt, Germany).

Table S1. Retention Time, Calibration Curves, and Coefficients of Determination of Quantified Substances using LC-MS/MS.

Analyte	Equation	R^2
	Polyphenols	
gentisic acid	$y = 6.13482e^5 x + (-5.60790e^4)$	0.9987
protocatechuic acid	$y = 1.91507 x + (-14936.77590)$	0.9924
p-hydroxybenzoic acid	$y = 9785.16236 x + 8963.75107$	0.9886
p-hydroxybenzaldehyde	$y = 7.68249e^5 x + 17612.61178$	0.9991
ferulic acid	$y = 11993.34482 x + 3.22923e^4$	0.9900
phloretic acid	$y = 606.55521 x + 425.31500$	0.9977
dihydrocaffeic acid	$y = 6.58189e^4x + (-16325.27870)$	0.9935
quercetin-3-*O*-glucuronide	$y = 1.73402e^5 x + 1893.96969$	0.9932
methyl gallate	$y = 2.32436e^6 x + (-13823.99201)$	0.9983
myricetin-3-*O*-glucoside	$y = 3.0456e^5 x + (-1.06153e^5)$	0.9913
naringenin	$y = 8.78326e^5 x + 9471.19479$	0.9917
isorhamnetin	$y = 3.13467e^6 x + 4.29466e^5$	0.9924
quercetin	$y = 3.22430e^5 x + 7.84377e^4$	0.9981
syringetin	$y = 2.61540e^6 x + 3.14102e^5$	0.9941
eriodictyol	$y = 1.24057e^6 x + (-7.43749e^4)$	0.9988
(-)-catechin gallate	$y = 8.21956e^5x + (-1.34637e^5)$	0.9983
(-)-epicatechingallate	$y = 5.24603e^5x + (-1.28814e^5)$	0.9985
(-)-epigallocatechin	$y = 4.64464e^5x + (-9.09384e^4)$	0.9992
(-)-gallocatechin	$y = 3.89231e^5x + (-7.74978e^4)$	0.9992
	Organic acids after 3-NPH derivatization	
succinic acid	$y = 0.08826 x + (-0.00509)$	0.9960
acetic acid	$y = 0.02351 x + 0.00911$	0.9933
lactic acid	$y = 0.01513x + 0.00320$	0.9952
oxalic acid	$y = 0.05907x + 0.00254$	0.9965
	Anthocyanins/Anthocyanidins	
pelargonidin	$y = 1.10314e^6 x + (-8.28027e^6)$	0.9824

cyanidin	$y = 7.9954e^{5} x + (-4.20492e^{6})$	0.9936
peonidin	$y = 2.41756e^{6} x + (-1.40403e^{7})$	0.9913
delphinidin	$y = 1.13318e^{6} x + (-5.18693e^{6})$	0.9959
petunidin	$y = 2.28557e^{6} x + (-1.03630e^{7})$	0.9946
malvidin	$y = 4.61919e^{5} x + 3.12095e^{5}$	0.9919
cyanidin-3-*O*-glc	$y = 2.17090e^{7} x + 5.46041e^{6}$	0.9867
peonidin-3-*O*-glc	$y = 2.22866e^{7} x + 6.01124e^{6}$	0.9878
delphinidin-3-*O*-glc	$y = 2.82693e^{7} x + 9.97647e^{6}$	0.9857
petunidin-3-*O*-glc	$y = 2.09017e^{7} x + 3.85221e^{6}$	0.9888
malvidin-3-*O*-glc	$y = 2.20845e^{7} x + 4.26985e^{6}$	0.9888

Table S2. Validation Experiments for Quantitation of Chemical Degradation Products after Alkaline Hydrolysis using LC-MS/MS.

analyte	RSD intraday[a] (%)	RSD interday[b] (%)	recovery (%)
	Polyphenols		
gallic acid	23	28	70
caffeic acid	8	9	85
p-coumaric acid	4	11	94
syringic acid	7	13	74
vanillic acid	6	11	102
gentisic acid	4	13	94
protocatechuic acid	5	8	107
p-hydroxybenzoic acid	2	3	94
p-hydroxybenzaldehyde	2	13	77
ferulic acid	25	28	103
phloretic acid	7	7	84
dihydrocaffeic acid	10	10	74
quercetin-3-*O*-glucuronide	2	7	101
syringetin-3-*O*-glc	20	20	92
quercetin-3-*O*-gal	12	47	95
(+)-catechin	5	19	83
	Organic acids after 3-NPH derivatization		
tartaric acid	3	14	113
citric acid	6	14	110
malic acid	8	23	106
acetic acid	8	10	78
succinic acid	4	7	110
lactic acid	4	15	96
oxalic acid	8	11	120

[a] Relative Standard Deviation of Intraday Precision. Five aliquots of HMW samples were analyzed for the compounds on the same day. [b] Relative Standard Deviation of Interday Precision. Five aliquots of HMW samples were analyzed for the compounds on consecutive days.

Table S3. Validation Experiments for Quantitation of Chemical Degradation Products after Thiolytic Depolymerization by means of LC-MS/MS.

analyte	RSD intraday[a] (%)	RSD interday[b] (%)	recovery (%)
	Polyphenols		
gallic acid	6	6	89
p-coumaric acid	11	15	87
gentisic acid	1	2	86
phloretic acid	17	17	92
protocatechuic acid	14	17	82
gallic acid ethyl ester	1	13	98
t-caffeic acid ethyl ester	12	12	100
methyl gallate	11	14	115
myricetin-3-*O*-glc	18	17	91
naringenin	8	9	91
isorhamnetin	4	11	81
quercetin	3	10	96
syringetin	8	4	104
quercetin-3-*O*-glucuronide	2	5	95
eriodictyol	5	6	96
syringetin-3-*O*-glc	9	11	93
quercetin-3-*O*-gal	18	18	84
p-coumaric acid ethyl ester	2	4	88
(+)-catechin	6	8	109
(-)-epicatechin	10	12	110
catechin gallate	3	6	84
epicatechin gallate	3	4	86
epigallocatechin	2	5	114
gallocatechin	6	8	93
	Anthocyanins/Anthocyanidins		
pelargonidin	0	9	116

cyanidin	7	9	120
peonidin	7	4	117
delphinidin	5	7	110
petunidin	5	11	115
malvidin	2	14	112
cyanidin-3-*O*-glc	1	5	97
peonidin-3-*O*-glc	3	10	79
delphinidin-3-*O*-glc	3	19	99
petunidin-3-*O*-glc	3	10	84
malvidin-3-*O*-glc	1	7	105

[a] Relative Standard Deviation of Intraday Precision. Five aliquots of HMW samples were analyzed for the compounds on the same day. [b] Relative Standard Deviation of Interday Precision. Five aliquots of HMW samples were analyzed for the compounds on consecutive days.

Table S4. LOD and LOQ of Chemical Degradation Products using LC-MS/MS.

analyte	LOD[a] (µmol/L)	LOQ[b] (µmol/L)
	Polyphenols	
gentisic acid	0.001	0.005
protocatechuic acid	0.0002	0.008
p-hydroxybenzoic acid	0.0002	0.017
p-hydroxybenzaldehyde	0.003	0.008
ferulic acid	0.023	0.049
phloretic acid	0.366	1.465
dihydrocaffeic acid	0.005	0.052
quercetin-3-*O*-glucuronide	0.002	0.020
methyl gallate	0.004	0.009
myricetin-3-*O*-glc	0.012	0.136
naringenin	0.0003	0.001
isorhamnetin	0.0003	0.0004
quercetin	0.005	0.007
syringetin	0.0002	0.0003
eriodictyol	0.0001	0.003
catechin gallate	0.001	0.006
epicatechin gallate	0.003	0.006
epigallocatechin	0.003	0.006
gallocatechin	0.006	0.023
	Organic acids after 3-NPH derivatization	
acetic acid	0.003	0.034
succinic acid	0.003	0.024
lactic acid	0.004	0.005
oxalic acid	0.002	0.034
	Anthocyanins/Anthocyanidins	
pelargonidin	0.0003	0.0120
cyanidin	0.0018	0.1286
peonidin	0.0008	0.0029

delphinidin	0.0262	0.2338
petunidin	0.0042	0.0122
malvidin	0.0005	0.0044
cyanidin-3-*O*-glc	0.0003	0.0006
peonidin-3-*O*-glc	0.00006	0.00013
delphinidin-3-*O*-glc	0.0019	0.0038
petunidin-3-*O*-glc	0.0001	0.0002
malvidin-3-*O*-glc	0.0001	0.0002

[a] Limit of Detection. [b] Limit of Quantitation.

Table S5. MRM transitions of analyzed compounds.

analyte	Q1	Q3	RT [min]	DP	EP	CE	CXP
			Polyphenols				
gentisic acid	152.902	107.9	2.08	-30	-10	-28	-13
protocatechuic acid	152.902	107.9	1.37	-45	-10	-32	-11
p-hydroxybenzoic acid	136.9	65.0	2.06	-20	-10	-40	-9
p-hydroxybenzaldehyde	120.914	92.0	3.47	-15	-10	-34	-13
ferulic acid	192.9	134.0	7.26	-25	-10	-22	-15
phloretic acid	164.922	59.1	4.40	-105	-10	-16	-1
dihydrocaffeic acid	181.0	108.9	2.44	-50	-10	-20	-13
quercetin-3-*O*-glucuronide	476.9	178.9	7.84	-85	-10	-42	-17
methyl gallate	182.936	123.9	2.45	-50	-10	-28	-13
myricetin-3-*O*-glucoside	479.0	270.7	6.41	-195	-10	-50	-27
naringenin	270.8	151.2	12.82	-35	-10	-24	-17
isorhamnetin	314.936	299.9	13.41	-45	-10	-30	-33
quercetin	300.941	150.9	10.19	-95	-10	-28	-17
syringetin	344.979	314.9	13.48	-90	-10	-34	-35
eriodictyol	287.0	135.0	11.65	-30	-10	-34	-15
catechin gallate	440.955	168.9	8.49	-40	-10	-24	-11
epicatechin gallate	440.905	289.1	8.21	-70	-10	-22	-7
epigallocatechin	304.977	124.9	1.89	-70	-10	-28	-13
gallocatechin	304.990	124.9	1.12	-45	-10	-28	-15
			Organic acids after 3-NPH derivatization				
succinic acid	251.983	233.9	2.25	-30	-10	-18	-29
succinic acid-$^{13}C_2$	254.025	235.9	2.24	-50	-10	-18	-27
acetic acid	193.967	151.9	2.32	-60	-10	-18	-17

acetic acid-$^{13}C_2$	196.025	151.9	2.31	-45	-10	-18	-15
lactic acid	223.903	151.9	2.20	-70	-10	-20	-17
lactic acid-$^{13}C_2$	227.028	151.9	2.20	-5	-10	-20	-15
oxalic acid	223.939	152.1	1.81	-5	-10	-14	-17
oxalic acid-$^{13}C_2$	225.886	151.9	1.81	-5	-10	-14	-15
		Anthocyanins/Anthocyanidins					
pelargonidin	270.975	120.9	6.65	1	10	45	14
cyanidin	287.014	213.0	5.85	1	10	43	24
peonidin	300.990	286.1	7.00	76	10	35	28
delphinidin	302.975	229.0	5.00	1	10	45	24
petunidin	316.973	302.1	6.16	66	10	35	32
malvidin	330.993	315.0	7.20	21	10	41	34
cyanidin-3-*O*-glc	448.982	287.0	4.50	1	10	31	16
peonidin-3-*O*-glc	463.040	301.0	5.33	1	10	33	16
delphinidin-3-*O*-glc	464.975	303.1	4.07	26	10	29	24
petunidin-3-*O*-glc	479.003	317.0	4.80	26	10	27	18
malvidin-3-*O*-glc	493.061	330.9	5.57	61	10	67	26

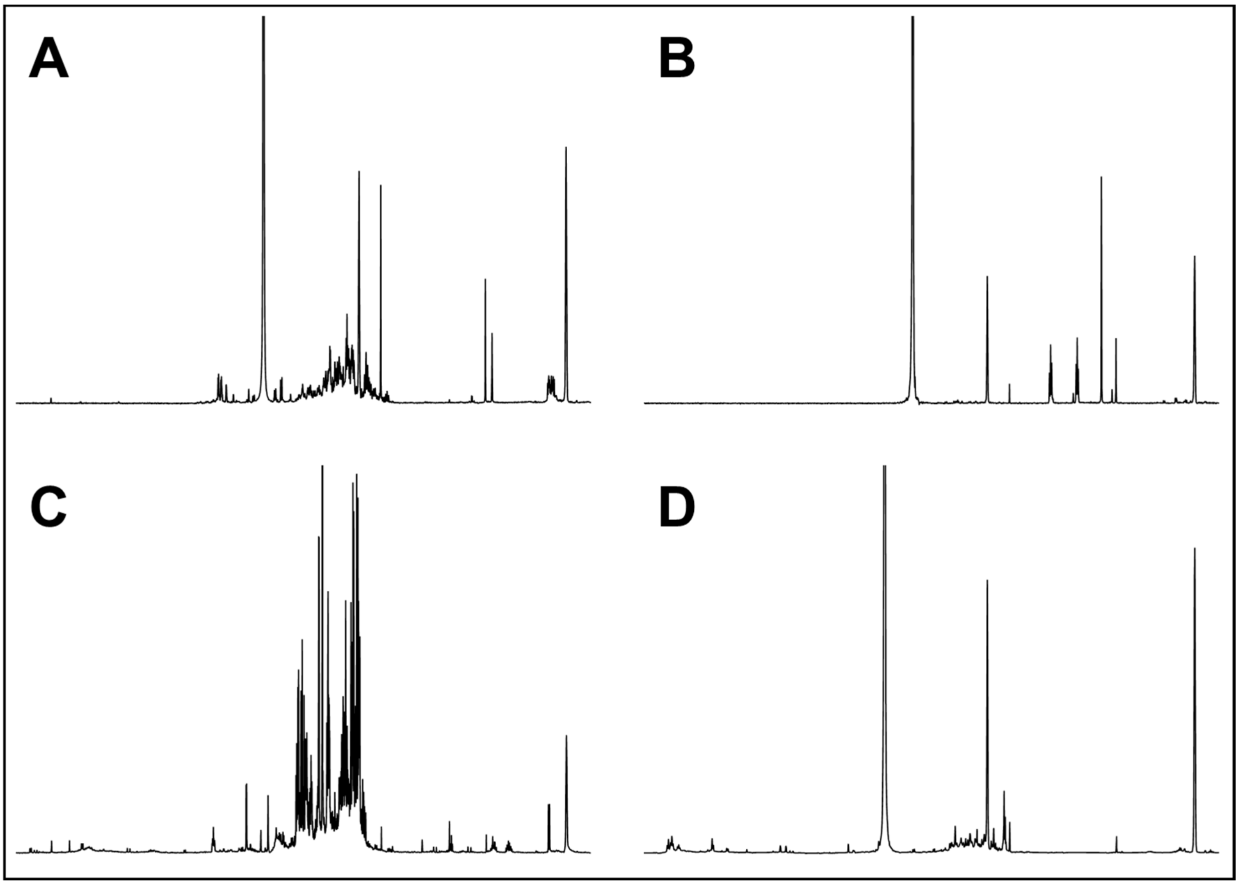

Figure S1. Excerpts of ^{1}H NMR spectra (1-8 ppm; 500 MHz; ethanol-d_6/D_2O, 14/86, v/v; pH 3.8; 298 K) of low molecular weight fractions <5 kDa after chemical degradation (A: acidic hydrolysis (H_2SO_4) 2h 100°C; B: acidic hydrolysis (HCl); C: alkaline hydrolysis 2h 60 °C; D: thiolytic depolymerization 2h 60 °C).

Table S6. Concentrations of Chemical Degradation Products of Hydrolysates of Red Wine Polymers.

**compound**	**concentration [µg/mg HMW]**								
	**>50 kDa**			**30-50 kDa**			**5-30 kDa**		
	phenolic compounds after alkaline hydrolysis								
2h	*40 °C*	*60 °C*	*80 °C*	*40 °C*	*60 °C*	*80 °C*	*40 °C*	*60 °C*	*80 °C*
gallic acid	0.10	0.08	0.01	0.39	0.06	n.d.	0.21	0.01	0.03
caffeic acid	0.02	0.07	0.02	0.15	0.01	0.09	0.18	0.12	0.03
p-coumaric acid	3.32	3.08	2.99	4.78	4.30	4.53	4.75	3.57	3.62
syringic acid	11.89	18.86	20.70	19.24	23.81	35.30	22.10	22.41	27.70
vanillic acid	2.42	2.71	6.78	1.37	3.57	6.43	2.41	1.70	6.35
gentisic acid	<0.01	<0.01	<0.01	<0.01	<0.01	<0.01	<0.01	<0.01	<0.01
protocatechuic acid	1.06	0.97	0.56	1.53	1.31	1.60	1.40	1.48	0.38
p-hydroxybenzoic acid	0.07	0.08	0.12	0.09	0.12	0.12	0.09	0.09	0.09
p-hydroxybenzaldehyde	0.15	0.15	0.12	0.05	0.07	0.09	0.09	0.09	0.07
ferulic acid	0.04	0.06	0.10	0.03	0.05	0.11	0.08	<0.01	0.07
phloretic acid	0.03	0.02	0.05	<0.01	0.02	0.03	0.01	<0.01	0.01
dihydrocaffeic acid	0.02	0.09	0.08	0.01	0.02	0.09	0.03	0.13	0.14
quercetin-3-*O*-glu	0.07	0.09	0.04	0.09	0.06	0.09	0.13	0.11	0.04
syringetin-3-*O*-glc	0.01	0.01	<0.01	0.01	0.01	0.01	0.02	0.02	0.02
quercetin-3-*O*-gal	0.01	0.01	0.01	0.01	0.01	0.01	0.01	0.01	0.01
**total content**	**19.21**	**26.28**	**31.58**	**27.73**	**33.42**	**48.50**	**31.48**	**29.74**	**38.56**
	organic acids after alkaline hydrolysis								
2h	*40 °C*	*60 °C*	*80 °C*	*40 °C*	*60 °C*	*80 °C*	*40 °C*	*60 °C*	*80 °C*
tartaric acid	2.13	1.99	2.53	0.40	1.08	1.25	2.24	3.89	4.02
succinic acid	1.02	1.81	2.80	0.67	1.42	2.24	1.20	2.59	3.76
acetic acid	7.03	9.46	15.61	6.80	12.56	16.77	7.28	11.56	19.46
lactic acid	10.34	13.11	22.90	5.11	7.38	14.57	12.58	18.32	29.23
malic acid	2.83	2.68	5.49	1.79	4.55	6.92	6.94	8.61	9.46
oxalic acid	0.64	0.23	0.46	0.14	0.05	0.19	0.54	0.26	0.48
citric acid	0.23	0.35	0.33	0.30	0.28	0.22	n.d.	n.d.	n.d.
(*E*)-aconitic acid	0.44	0.30	0.10	0.58	0.77	0.41	0.46	0.21	0.28
(*Z*)-aconitic acid	0.06	1.13	0.71	1.89	1.47	1.21	0.76	1.20	0.75
galacturonic acid	n.d.	n.d.	1.51	0.10	0.63	2.21	<0.01	0.31	1.88
**total content**	**24.72**	**31.06**	**52.44**	**17.78**	**30.19**	**45.99**	**32.01**	**46.64**	**69.32**
	flavan-3-ols after alkaline hydrolysis								
2h	*40 °C*	*60 °C*	*80 °C*	*40 °C*	*60 °C*	*80 °C*	*40 °C*	*60 °C*	*80 °C*
(+)-catechin	n.d.	0.02	n.d.	0.31	n.d.	0.03	0.11	0.12	0.10
(-)-epicatechin	n.d.	n.d.	n.d.	0.01	n.d.	n.d.	n.d.	n.d.	n.d.
**total content**	**n.d.**	**0.02**	**n.d.**	**0.31**	**n.d.**	**0.03**	**0.11**	**0.12**	**0.10**
	amino acids after acidic hydrolysis (HCl)								
alanine		0.73			0.44			1.12	
arginine		0.42			0.27			0.38	

aspartic acid	2.47	3.71	3.27
glutamic acid	0.99	0.65	1.40
glutamine	0.59	0.41	0.39
glycine	0.44	0.40	1.16
histidine	0.34	0.17	0.14
isoleucine	0.15	0.07	0.13
leucine	0.12	0.03	0.19
lysine	0.46	0.34	0.31
phenylalanine	0.20	0.03	0.23
pyroglutamic acid	0.33	0.28	0.26
serine	2.15	1.08	0.87
threonine	0.84	0.39	0.42
**total content**	**10.23**	**8.27**	**10.27**

carbohydrates and polyols after acidic hydrolysis (H_2SO_4)

	2h 100 °C	*8h 100 °C*	*2h 100 °C*	*8h 100 °C*	*2h 100 °C*	*8h 100 °C*
galactose	23.51	85.09	36.18	82.31	33.63	49.29
mannose	21.71	29.19	13.21	24.58	12.16	15.87
arabinose	16.39	21.26	9.98	17.24	7.45	9.57
rhamnose	8.27	11.17	5.80	11.08	11.93	16.91
glucose	4.55	7.80	4.25	9.94	12.45	18.89
mannitol	0.43	0.72	0.45	0.81	0.76	1.02
glycerol	1.49	2.05	0.36	0.79	0.74	1.17
sorbitol	0.24	0.31	0.07	0.22	0.05	0.12
trehalose	0.26	0.33	n.d.	n.d.	n.d.	n.d.
**total content**	**76.85**	**157.92**	**70.3**	**146.97**	**79.17**	**112.84**

flavan-3-ols after thiolysis

	2h 40 °C	*2h 60 °C*	*2 min 90 °C*	*2h 40 °C*	*2h 60 °C*	*2 min 90 °C*	*2h 40 °C*	*2h 60 °C*	*2 min 90 °C*
(+)-catechin	3.52	2.26	7.68	4.94	3.15	7.08	7.75	4.50	11.79
(-)-epicatechin	1.89	1.00	4.31	2.89	1.47	4.35	4.70	2.29	8.12
catechin gallate	0.01	0.03	0.02	0.01	0.04	0.02	0.01	0.03	0.01
epicatechin gallate	0.18	0.16	0.45	0.22	0.21	0.36	0.13	0.13	0.26
epigallocatechin	0.12	0.06	0.25	0.15	0.08	0.15	0.14	0.08	0.19
gallocatechin	0.29	0.15	0.52	0.36	0.19	0.32	0.33	0.16	0.36
**total content**	**6.01**	**3.66**	**13.23**	**8.57**	**5.14**	**12.28**	**13.06**	**7.19**	**20.73**

phenolic compounds after thiolysis

	2h 40 °C	*2h 60 °C*	*2 min 90 °C*	*2h 40 °C*	*2h 60 °C*	*2 min 90 °C*	*2h 40 °C*	*2h 60 °C*	*2 min 90 °C*
gallic acid	0.02	0.02	n.d.	n.d.	n.d.	0.02	n.d.	n.d.	0.01
p-coumaric acid	n.d.	0.09	0.09	0.07	0.10	0.09	0.09	0.10	0.09
gentisic acid	0.01	0.01	0.01	0.01	0.01	0.01	0.01	0.01	<0.01
phloretic acid	0.01	0.02	0.01	0.05	<0.01	0.02	0.02	0.01	<0.01
protocatechuic acid	n.d.	n.d.	n.d.	n.d.	n.d.	0.01	0.01	<0.01	0.02
gallic acid ethyl ester	0.01	0.01	<0.01	n.d.	n.d.	<0.01	n.d.	n.d.	0.01

t-caffeic acid ethyl ester	0.01	0.01	0.01	n.d.	n.d.	n.d.	n.d.	n.d.	n.d.
methyl gallate	0.17	0.22	0.15	0.14	0.26	0.16	0.09	0.12	0.04
myricetin-3-*O*-glc	0.07	n.d.	0.07	0.06	n.d.	0.07	n.d.	n.d.	n.d.
naringenin	<0.01	<0.01	<0.01	<0.01	<0.01	<0.01	<0.01	<0.01	<0.01
isorhamnetin	<0.01	<0.01	<0.01	<0.01	<0.01	<0.01	0.01	0.01	<0.01
quercetin	<0.01	0.01	0.01	<0.01	0.01	0.01	0.03	0.04	0.03
syringetin	0.01	0.02	<0.01	0.01	0.02	0.01	0.03	0.05	0.01
quercetin-3-*O*-glu	<0.01	<0.01	0.01	0.01	0.01	0.01	<0.01	<0.01	<0.01
eriodictyol	0.01	0.01	0.01	0.01	0.01	0.01	0.01	0.01	0.01
syringetin-3-*O*-glc	<0.01	n.d.	0.01	<0.01	n.d.	<0.01	<0.01	<0.01	0.01
quercetin-3-*O*-gal	0.02	n.d.	0.02	0.02	n.d.	0.02	0.02	n.d.	0.02
p-coumaric acid ethyl ester	0.03	0.02	0.01	n.d.	n.d.	n.d.	n.d.	n.d.	n.d.
**total content**	**0.37**	**0.44**	**0.41**	**0.38**	**0.42**	**0.44**	**0.32**	**0.35**	**0.25**
anthocyanins/anthocyanidins after thiolysis									
	2h 40 °C	*2h 60 °C*	*2 min 90 °C*	*2h 40 °C*	*2h 60 °C*	*2 min 90 °C*	*2h 40 °C*	*2h 60 °C*	*2 min 90 °C*
pelargonidin	0.93	0.98	0.87	0.82	0.89	0.90	0.94	0.89	0.86
cyanidin	0.82	0.99	0.76	0.79	1.06	0.86	1.01	1.13	0.83
peonidin	0.90	1.10	0.87	0.84	1.09	0.97	1.34	1.60	1.09
delphinidin	0.70	0.82	0.65	0.64	0.81	0.73	0.90	1.00	0.75
petunidin	0.65	0.77	0.62	0.59	0.75	0.69	0.98	1.19	0.85
malvidin	1.59	2.81	2.67	1.87	2.98	4.68	8.28	10.17	7.92
cyanidin-3-*O*-glc	0.04	0.09	0.09	0.05	0.10	0.18	0.37	0.48	0.37
peonidin-3-*O*-glc	0.32	0.57	0.55	0.41	0.66	0.94	1.65	2.04	1.59
delphinidin-3-*O*-glc	0.04	0.09	0.10	0.05	0.11	0.25	0.53	0.68	0.53
petunidin-3-*O*-glc	0.30	0.51	0.50	0.36	0.56	0.95	1.77	2.17	1.70
malvidin-3-*O*-glc	2.46	3.98	3.79	2.79	4.27	6.01	9.38	10.18	7.58
**total content**	**8.74**	**12.70**	**11.46**	**9.20**	**13.26**	**17.15**	**27.14**	**31.53**	**24.09**

Table S7. UHPLC-ToF-MS Data of HMW fraction >5 kDa after alkaline hydrolysis in ESI- mode.

compound	neutral mass [Da]	formula	adduct	fragment [m/z]	expected fragment [m/z]	RT [min]	expected RT [min]
caffeic acid	180.0423	$C_9H_8O_4$	[M-H]	179.0343	179.0610	2.03	2.03
dihydrocaffeic acid	182.0579	$C_9H_{10}O_4$	[M-H]	181.0139	181.0511	2.01	2.00
gallic acid	170.0215	$C_7H_6O_5$	[M-H]	169.0114	169.0143	1.45	1.48
malic acid	134.0215	$C_4H_6O_5$	[M-H]	133.0142	133.0139	1.03	1.03
p-coumaric acid	164.0473	$C_9H_8O_3$	[M-H]	199.0501	199.0504	2.27	2.27
p-hydroxybenzaldehyde	122.0368	$C_7H_6O_2$	[M-H]	121.0288	121.0304	2.30	2.29
p-hydroxybenzoic acid	138.0317	$C_7H_6O_3$	[M-H]	137.0241	137.0242	2.00	1.98
phloretic acid	166.0630	$C_9H_{10}O_3$	[M-H]	165.0587	165.0561	2.22	2.21
vanillic acid	168.0423	$C_8H_8O_4$	[M-H]	167.0350	167.0346	2.09	2.09

Table S8. UHPLC-ToF-MS Data of HMW fraction >5 kDa after thiolytic depolymerization in ESI$^-$ mode.

compound	neutral mass [Da]	formula	adduct	fragment [m/z]	expected fragment [m/z]	RT [min]	expected RT [min]
catechin	290.0790	$C_{15}H_{14}O_6$	[M-H]	289.0721	289.0759	1.87	1.87
catechin gallate	442.0900	$C_{22}H_{18}O_{10}$	[M-H]	441.0837	441.0852	2.19	2.19
epicatechin	290.0790	$C_{15}H_{14}O_6$	[M-H]	289.0722	289.0759	1.97	1.97
epicatechin gallate	442.0900	$C_{22}H_{18}O_{10}$	[M-H]	441.0506	441.0820	2.09	2.13
epigallocatechin	306.0740	$C_{15}H_{14}O_7$	[M-H]	305.0674	305.0696	1.74	1.74
eriodictyol	288.0634	$C_{15}H_{12}O_6$	[M-H]	287.0564	287.0599	2.72	2.73
methyl gallate	184.0372	$C_8H_8O_5$	[M-H]	183.0301	183.0346	1.97	1.97
gallocatechin	306.0740	$C_{15}H_{14}O_7$	[M-H]	305.0658	305.0692	1.62	1.62
isorhamnetin	316.0583	$C_{16}H_{12}O_7$	[M-H]	315.0878	315.0562	3.07	3.02
naringenin	272.0685	$C_{15}H_{12}O_5$	[M-H]	271.0606	271.0654	2.96	2.96
quercetin	302.0427	$C_{15}H_{10}O_7$	[M-H]	331.0357	331.0399	2.71	2.71
quercetin-3-*O*-*β*-D-galactoside	464.0955	$C_{21}H_{20}O_{12}$	[M-H]	463.0990	463.0923	2.14	2.14
syringetin	346.0689	$C_{17}H_{14}O_8$	[M-H]	345.0618	345.0652	2.97	2.98

Table S9. UHPLC-ToF-MS Data of HMW fraction >5 kDa after thiolytic depolymerization in ESI+ mode.

compound	neutral mass [Da]	formula	adduct	fragment [m/z]	expected fragment [m/z]	RT [min]	expected RT [min]
cyanidin	287.0550	$C_{15}H_{11}O_6^+$	[M+]	287.0555	287.0615	1.98	1.97
malvidin	331.0812	$C_{17}H_{15}O_7^+$	[M+]	331.0823	331.0823	2.16	2.11
malvidin-3-*O*-glucoside	493.1341	$C_{23}H_{25}O_{12}^+$	[M+]	493.1355	493.1376	1.82	1.82
peonidin	301.0707	$C_{16}H_{13}O_6^+$	[M+]	301.0720	301.0771	2.16	2.15
peonidin-3-*O*-glucoside	463.1235	$C_{22}H_{23}O_{11}^+$	[M+]	463.1244	463.1300	1.82	1.81
petunidin-3-*O*-glucoside	479.1184	$C_{22}H_{23}O_{12}^+$	[M+]	479.1194	479.1221	1.72	1.71

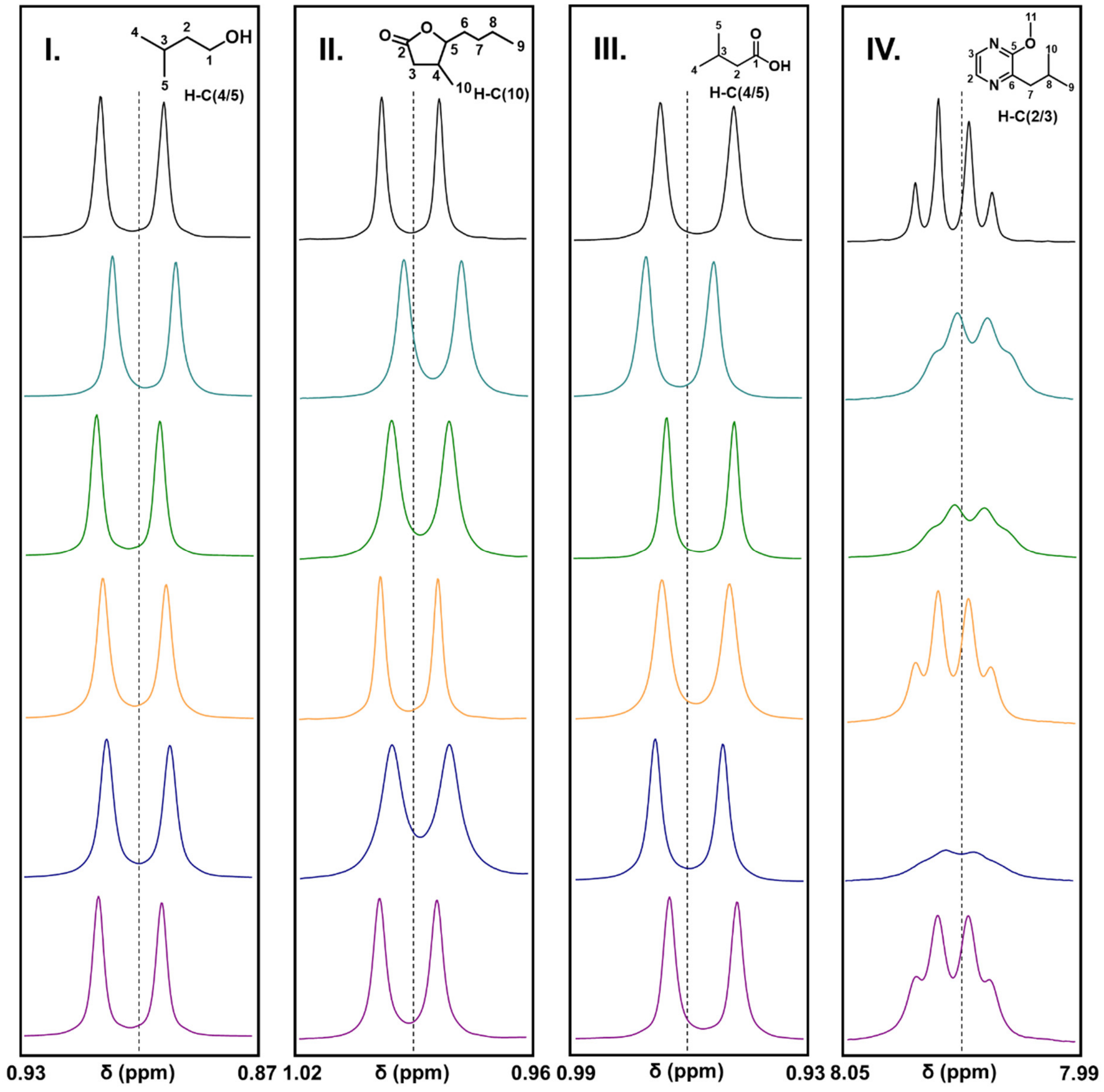

Figure S2. Excerpts of qHNMR spectra (400 MHz; ethanol-d_6/D_2O, 14/86, v/v; pH 3.8; 298 K) of NMR-based interaction studies of 3-methyl-1-butanol (I), cis-whisky lactone (II), 3-methylbutanoic acid (III) and 3-isobutyl-2-methoxypyrazine (IV) with HMW fractions after chemical degradation of HMW fraction >50 kDa (control solution without HMW (black), native HMW (light blue), HMW after alkaline hydrolysis (green), HMW after acidic hydrolysis with H_2SO_4 (orange), HMW after thiolytic depolymerization (dark blue), and HMW after acidic hydrolysis with HCl (purple)) in equal concentration (2.89 g/L) after 30 min of incubation at RT.

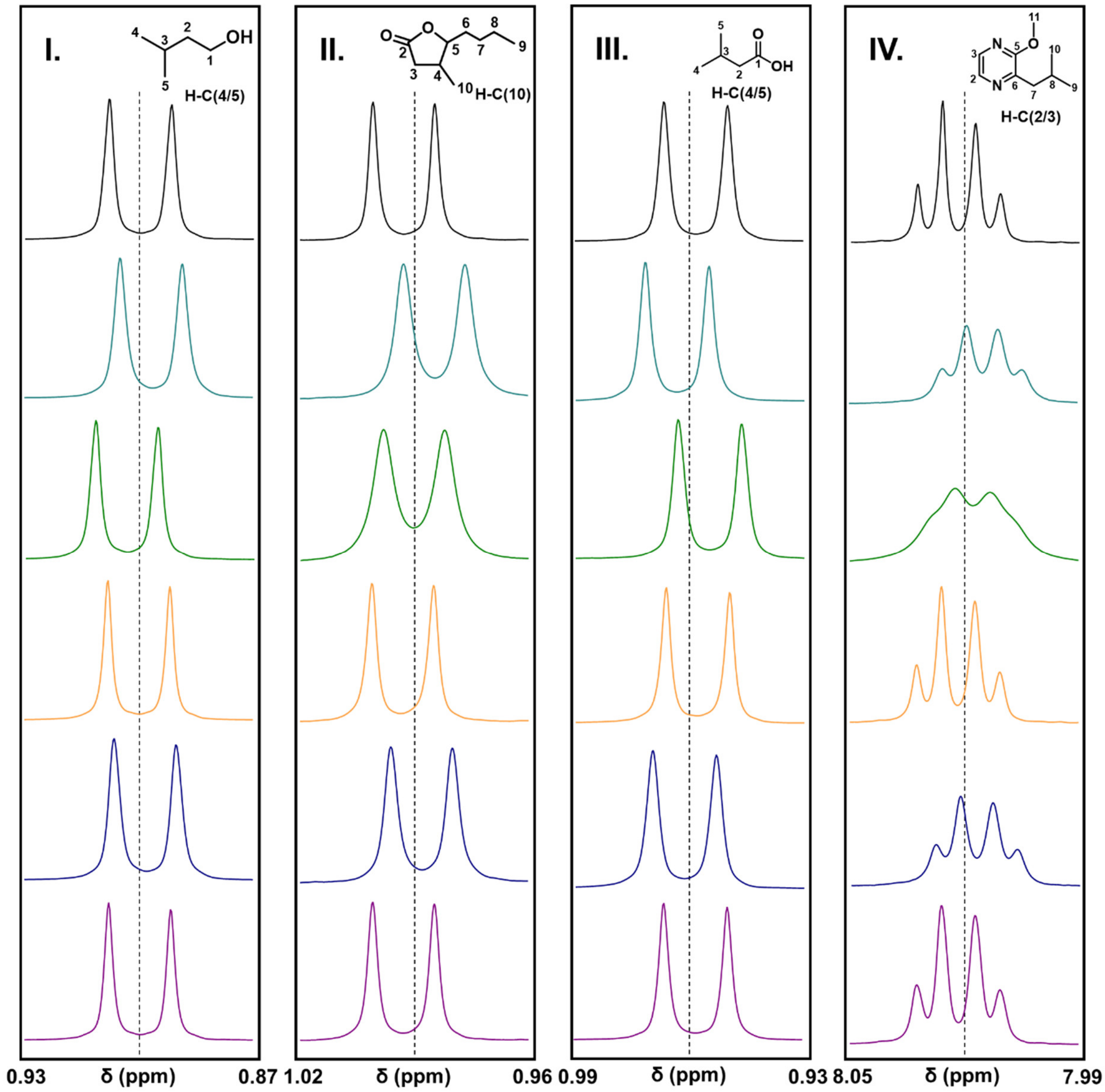

Figure S3. Excerpts of qHNMR spectra (400 MHz; ethanol-d_6/D_2O, 14/86, v/v; pH 3.8; 298 K) of NMR-based interaction studies of 3-methyl-1-butanol (I), cis-whisky lactone (II), 3-methylbutanoic acid (III) and 3-isobutyl-2-methoxypyrazine (IV) with HMW fractions after chemical degradation of HMW fraction 5–30 kDa (control solution without HMW (black), native HMW (light blue), HMW after alkaline hydrolysis (green), HMW after acidic hydrolysis with H_2SO_4 (orange), HMW after thiolytic depolymerization (dark blue), and HMW after acidic hydrolysis with HCl (purple)) in equal concentration (2.89 g/L) after 30 min of incubation at RT.
